# A two-step strategy for identification of plasma protein biomarkers for endometrial and ovarian cancer

**DOI:** 10.1186/s12014-018-9216-y

**Published:** 2018-12-01

**Authors:** Stefan Enroth, Malin Berggrund, Maria Lycke, Martin Lundberg, Erika Assarsson, Matts Olovsson, Karin Stålberg, Karin Sundfeldt, Ulf Gyllensten

**Affiliations:** 1 Department of Immunology, Genetics, and Pathology, Biomedical Center, Science for Life Laboratory (SciLifeLab) Uppsala, Uppsala University, Box 815, 75108 Uppsala, Sweden; 2 OLINK Proteomics, Uppsala Science Park, 75183 Uppsala, Sweden; 3 Department of Women’s and Children’s Health, Uppsala University, Uppsala, Sweden; 4 Department of Obstetrics and Gynecology, Institute of Clinical Sciences, Sahlgrenska Academy at Gothenburg University, Gothenburg, Sweden

**Keywords:** Ovarian cancer, Endometrial cancer, Proximity extension assay (PEA), Sensitivity, Specificity, Diagnostics

## Abstract

**Background:**

Over 500,000 women worldwide are diagnosed with ovarian or endometrial cancer each year. We have used a two-step strategy to identify plasma proteins that could be used to improve the diagnosis of women with an indication of gynecologic tumor and in population screening.

**Methods:**

In the discovery step we screened 441 proteins in plasma using the proximity extension assay (PEA) and five Olink Multiplex assays (CVD II, CVD III, INF I, ONC II, NEU I) in women with ovarian cancer (n = 106), endometrial cancer (n = 74), benign ovarian tumors (n = 150) and healthy population controls (n = 399). Based on the discovery analyses a set of 27 proteins were selected and two focused multiplex PEA assays were developed. In a replication step the focused assays were used to study an independent set of cases with ovarian cancer (n = 280), endometrial cancer (n = 228), women with benign ovarian tumors (n = 76) and healthy controls (n = 57).

**Results:**

In the discovery step, 27 proteins that showed an association to cancer status were identified. In the replication analyses, the focused assays distinguished benign tumors from ovarian cancer stage III–IV with a sensitivity of 0.88 and specificity of 0.92 (AUC = 0.92). The assays had a significantly higher AUC for distinguishing benign tumors from late stage ovarian cancer than using CA125 and HE4 (*p *= 9.56e−22). Also, population controls could be distinguished from ovarian cancer stage III–IV with a sensitivity of 0.85 and a specificity of 0.92 (AUC = 0.89).

**Conclusion:**

The PEA assays represent useful tools for identification of new biomarkers for gynecologic cancers. The selected protein assays could be used to distinguish benign tumors from ovarian and endometrial cancer in women diagnosed with an unknown suspicious pelvic mass. The panels could also be used in population screening, for identification of women in need of specialized gynecologic transvaginal ultrasound examination.

**Funding:**

The Swedish Cancer Foundation, Vinnova (SWELIFE), The Foundation for Strategic Research (SSF), Assar Gabrielsson Foundation.

**Electronic supplementary material:**

The online version of this article (10.1186/s12014-018-9216-y) contains supplementary material, which is available to authorized users.

## Introduction

In 2012 more than 500,000 women worldwide were diagnosed with epithelial ovarian cancer (OC) or endometrial cancer (EC) [1]. OC is the most lethal gynecologic malignancy, with 238,719 cases reported worldwide in 2012, corresponding to 3.4% of all cancer [1].

OC is an heterogenous disease with at least five sub-types. The biomarker CA125 can detect the most common late stage high-grade serous cancer, but lack diagnostic power for early stage and the less common ovarian adenocarcinomas, especially mucinous cancer. Also, CA125 often result in false positive results in inflammatory diseases such as endometriosis and is not regarded appropriate for fertile women, i.e. those aged 50 or below. The risk of ovarian malignancy algorithm (ROMA) is based on CA125, HE4 and menopausal status to assign women with adnexal ovarian mass into high-risk and low-risk groups. Cut-off for ROMA was estimated at a set specificity of 0.75 and has a sensitivity of 0.94 [2]. In the hands of specialists, transvaginal ultrasound (TVU) assessment can out-perform ROMA, but these specialized units are very scarce, while a serum test can be easily performed. The multivariate index assay Overa^®^, based on five plasma proteins, can distinguish between benign tumors and OC with a sensitivity of 0.69 and specificity of 0.91 [3]. Additional plasma biomarkers have been described but not yet clinically evaluated [4, 5]. The Risk of Ovarian Cancer Algorithm (ROCA) estimates the changes in annual CA125 measurements to identify women with high-risk scores in a screening population and refer these to specialized units for TVU examination. Use of ROCA followed by TVU has been shown to result in an increase in the number of women with OCs detected than using of a fixed cutoff for CA125, with half of these women detected by ROCA prior to CA125 > 35 and the other half at the same time as CA125 > 35 [6]. The United Kingdom Collaborative Trial of Ovarian Cancer Screening (UKCTOCS) has reported a reduction in ovarian cancer deaths, excluding prevalent cases, using annual screening using ROCA followed by TVU for ROCA positive subjects [7].

EC is the most frequent gynecologic malignancy in the developed world and in 2012 affected 319,605 women, corresponding to 7.1% of all cancer [1]. EC is diagnosed in early stage and have high 5-year survival due to early typical symptoms, such as post-menopause bleedings. Women with EC go through hysterectomy, bilateral salpingoophorectomy and after risk stratification, pelvic lymph node resection. Imaging techniques like CT, MRI and PET have shown variable performance in predicting the depth of EC, myometrial growth, cervical invasion and lymph node metastases [8]. The histopathology does not reliably reflect the underlying molecular nature of the tumors and more than 20% of tumors that were first classified as non-aggressive later develop into metastatic cancer [9]. Candidate plasma protein biomarkers have been described for EC, but in clinical practice preoperative biomarkers are lacking to stratify EC patients for lymph node resection, and to high-risk and low-risk groups for recurrence [10, 11].

For OC there is a strong need to identify proteins for differential diagnosis of suspicious ovarian tumors, early stage detection and sub-type specific diagnosis as complements to HE4 and CA125, in order to refer women to specific imaging techniques, reduce overtreatment and identify women with ovarian malignancy. For EC, biomarkers are needed for stratification of patients to different surgical interventions. In this study, we have used the proximity extension assay (PEA) to identify plasma proteins with a higher sensitivity and specificity that could be used to address the diagnostic needs for the two cancer types.

## Materials and methods

### Clinical material and ethics approval

The plasma samples of women with ovarian and endometrial tumors were from the UCAN collection at Uppsala Biobank, Uppsala University (UU), and the Gynecology tumor biobank at Sahlgrenska University Hospital (SU) (Table 1). All tumors were examined by a pathologist specialized in gynecologic cancers for histology, grade and stage according to International Federation of Gynecology and Obstetrics (FIGO) standards. Plasma samples from women with an indication of adnexal ovarian mass based on TVU and that received a diagnosis of benign conditions, including simple cysts (inclusion cysts, rete ovarii cysts, mesonephric cysts), follicle and corpora luteum cysts, endometriosis, teratoma and benign adenoma, were included as benign tumors. The plasma samples from healthy population controls were from women participating in The Northern Sweden Population Health Study (NSPHS) and from controls in the UCAN collection (Table 1). All plasma samples were frozen and stored at − 70 °C until used in the study.

**Table 1:** Baseline information for women included in the discovery and replication steps

	Discovery				Replication				
	Benign tumors cohort I	Ovarian cancer cohort I	Endometrial cancer cohort I	Population controls I	Benign tumors cohort II	Ovarian cancer cohort II	Ovarian cancer cohort III	Endometrial cancer cohort	Population controls II
Number of women	150 (SAL1)	106 (SAL1)	74 (SU1)	399 (NSPHS)	76 (SU2)	160 (SU2)	120 (UU)	228 (UU)	57 (UU)
*Age*									
Mean	59.8	61.2	59.84	49.3	60.8	61.8	60.9	66.8	57.3
Median	60	60	60	49	63	63	62	68	58
Range	16–88	28–88	29–86	14–94	22–88	19–87	21–86	29–90	18–86
SD	15.8	12.9	10.75974	20.3	14.5	11.9	12.9	10.5	14.2
P-value versus benign	NA	0.870	0.003665	2.22E−08	NA	0.868	0.937	0.001	0.163
Ethnicity	Caucasian	Caucasian	Caucasian	Caucasian	Caucasian	Caucasian	Caucasian	Caucasian	Caucasian
*Stage*									
I (I/IA/IB)		46	50 (0/41/9)			35	10	109 (3/83/23)	
II (II/IIA/IIB)		8	8 (8/0/0)			9	3	13 (12/0/1)	
III (III/IIIA/IIIB/IIIC/IIIC1/IIIC2)		47	10 (0/2/2/0/2/4)			96	40	22 (1/4/4/5/1/7)	
IV (IV/IVA/IVB)		5	6 (2/0/4)			19	24	11 (5/0/6)	
Classification unvailable						1	43	73	

### Plasma protein measurements

The abundance of 441 unique protein in plasma were analyzed using the Olink Multiplex assays CVD II, CVD III, INF I, ONC II and NEU I (http://www.olink.com) and quantified by real-time PCR using the Fluidigm BioMark™ HD real-time PCR platform as described earlier [12]. Briefly, for each protein a unique pair of oligonucleotide-labeled antibody probes bind to the targeted protein, and if the two probes are in close proximity a PCR target sequence is formed by a proximity-dependent DNA polymerization event and the resulting sequence is subsequently detected and quantified using standard real-time PCR. Data is then normalized and transformed using internal extension controls and inter-plate controls, to adjust for intra- and inter-run variation as described earlier [12]. The final assay read-out is given in Normalized Protein eXpression (NPX), which is an arbitrary unit on log2-scale where a high value corresponds to a higher protein expression. Each PEA measurement has a lower detection limit (LOD) calculated based on negative controls that are included in each run, and measurements below LOD were removed from further analysis. All assay characteristics including detection limits and measurements of assay performance and validations are available from the manufacturers webpage (http://www.olink.com). The analyses were based on 1 μL of plasma for each panel of 92 assays. To avoid batch effects, samples from the different disease entities and cohorts, including benign tumors, were randomized across assay plates. Each plate included internal controls, as described previously used to adjust for technical variation and/or sample irregularities.

### Development of focused multiplex PEA panels

The focused PEA assays were designed to be compatible with the Fluidigm 192 × 24 Integrated Fluidic Circuit (IFC) which can measure up to 24 assays on 192 samples simultaneously. Hence, 21 protein marker assays and three internal controls for run QC (Incubation Control, Extension Control and Detection control) can be analyzed per IFC. The main difference between the focused assays and the discovery assays is that the focused assays allow higher PEA probe concentration, which in turn means that higher levels of antigen can be measured without reaching the hook in the measuring range (i.e. too much antigen will decrease signals in a homogenous immunoassay format). This modification allowed inclusion of PEA assays from the CVD III panel, where samples are normally diluted 1:100 before analysis, into the focused assays used to analyze undiluted samples, making the differences in protein concentration to be measured more than 7 logs.

### Statistical methods

All calculations were carried out in R version 3.2.3 (R core team) [13]. Individual protein levels were also normalized by plate and sampling round using the MDimNormn-package [14]. This was done separately for the discovery and replication cohorts.

Significance levels for comparison of protein levels between cases and controls in the discovery cohort were calculated using the two-sided rank-based Spearman test (Wilcoxon). From the entire discovery-cohort, the top-ranking proteins for each of the two cancers was identified based on the *p* value in the comparison of benign tumors to cancer samples. 15 proteins were selected for association with OC and 16 with EC out of which 4 overlapped. Lasso and Elastic-Net Regularized Generalized Linear Models (’glmnet’ package in R) was used to fit multivariate models for each cancer using the selected proteins. These models were then evaluated based on the best point and their specificity at a fixed sensitivity of 0.95. This was repeated for each of the tumor/control combination investigated.

A few proteins overlapped between the two cancers, and a total of 27 proteins were selected to be characterized in the replication cohort using focused PEA-panels.

Model performance was evaluated by randomly splitting the observations in the replication cohort into a training set (75%) and a test set (25%). A model was then built using the training set and with the same proteins as selected from the discovery-cohort. The multivariate models were retrained in the replication cohorts using the ‘lm’ function in R. The reason for re-building the model in the replication data set is that the scaling of the NPX-values can differ between the multiplex PEA panels. This model was then used to predict the response variable in the test set. The random-split into training and test set will often result in different performance due to the limited sample-size. To accommodate for this, a cross-validation schema was applied and the process was repeated 100 times and model prediction errors on the training and test set were recorded. Sensitivity and specificity from the replication were reported as mean ± 1 SD of the 100 runs.

## Results

### Discovery step analyses

**Fig. 1:**
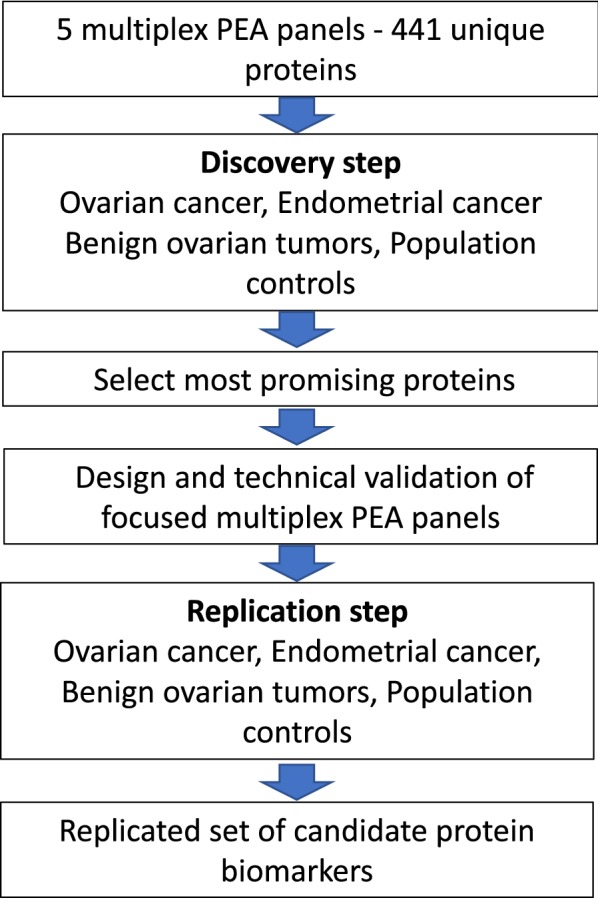
The study design for identification of protein biomarkers using PEA

The protein biomarker panels were identified using a two-step study design (Fig. 1). First, the levels of 441 individual proteins was measured in pretreatment plasma collected at time of diagnosis from women with OC or EC and compared to benign tumors and population controls (Table 1). No difference was found in the mean age of women with benign tumors and OC (stage I–IV) or between healthy controls and women with OC (Table 1). EC is diagnosed at a higher age than OC, which is reflected in the age difference seen between women with EC and those with benign tumors (Table 1).

**Fig. 2:**
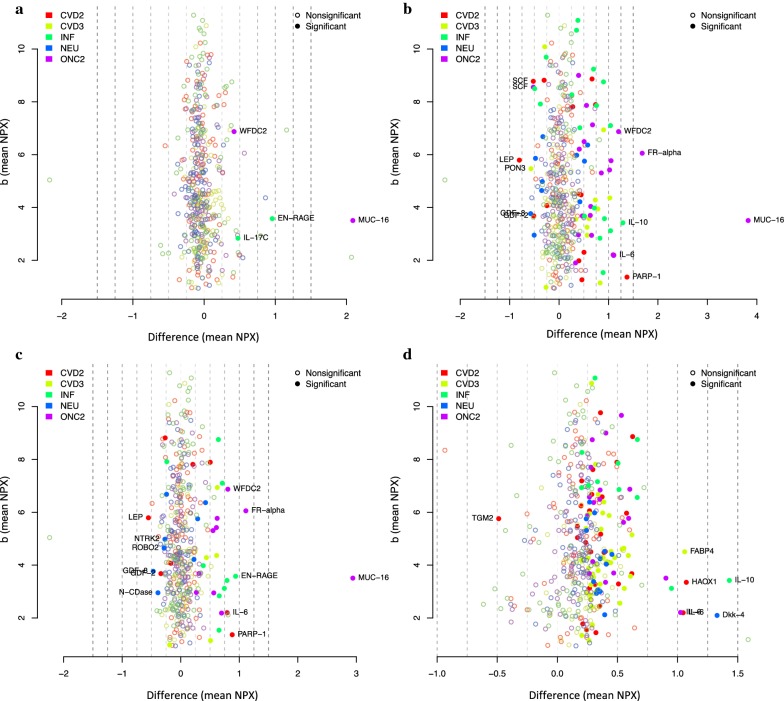
Distribution of differences in NPX values in the discovery step between benign tumors and OC (**a**–**c**) and between OC and EC (**d**). Only protein labels for the significant differences are shown

The distribution of differences in protein abundance (NPX-values) between women with OC or EC and benign tumors is shown for all proteins in Fig. 2. Proteins with a significant (Bonferroni-corrected significance threshold) difference in NPX values between benign tumors and OC (Fig. 2a–c) and between OC and EC (Fig. 2d) are labelled by name. The clinically used biomarker CA125 showed the largest differences in NPX value between OC cases and benign tumors. Nominal *p*-values for the comparisons of OC or EC with benign tumors and population controls are given for each protein in Additional file 1: Table 1.

Employing the approach described in the methods section, 27 of the 441 proteins in the discovery analysis were selected for further evaluation. Of these, 15 proteins (PRSS8, MK, WFDC2 (HE4), IL-10, SOD2, PARP-1, hK11, PVRL4, MUC-16 (CA125), FR-alpha, CDH3, NTRK3, IL-8, IL-17C, EN-RAGE) were selected from the comparison between OC stage I–IV and benign tumors (Table 2). Further, 16 proteins (PRSS8, MK, WFDC2 (HE4), IL-10, ADM, IDUA, MMP-7, FABP4, PCSK9, ST2, CTSZ, CCL16, Dkk-4, VEGF-A, IL-6, HGF) were selected from the comparison of EC with benign tumors (Table 3). Four of the proteins (PRSS8, MK, WFDC2, IL-10) were selected for both OC and EC.

**Table 2:** *p*-values for the individual proteins in the discovery and replication steps in the comparison of ovarian cancer to benign tumors, population controls

Uniprot no	Uniprot protein name	Proseek Protein ID	Cancer type	Ovarian cancer versus benign tumors						Ovarian cancer versus populations control			
				Discovery			Replication			Discovery			Replication
				Stage I–IV	Stage I–II	Stage III–IV	Stage I–IV	Stage I–II	Stage III–IV	Versus ovarian cancer stage I–IV	Versus ovarian cancer stage I–II	Versus ovarian cancer stage III–IV	Versus ovarian cancer stage I–IV
Q16651	Prostasin	CVD2_173_PRSS8	OE	1.08E−08	0.437395062	9.05E−11	3.95E−14	0.001859491	4.83E−16	0.028270178	1	1.29E−06	1.91E−05
P21741	Midkine	ONC2_161_MK	OE	2.85E−08	1	2.73E−13	2.60E−08	0.020068076	4.57E−09	0.007863784	1	6.50E−11	1.06E−06
Q14508	Major epididymis-specific protein E4	ONC2_182_WFDC2	OE	4.38E−14	0.012381315	5.28E−17	1.49E−21	7.55E−07	2.68E−23	2.11E−07	1	7.00E−14	2.35E−13
P22301	Interleukin-10	INF_162_IL-10	OE	1.64E−06	1	5.23E−10	0.479046977	1	0.097292101	1	0.00410586	0.529576635	1
P04179	Superoxide dismutase [Mn], mitochondrial	CVD2_137_SOD2	O	0.00066448	1	0.000688683	2.07E−09	0.042029761	8.56E−11	1	1	0.25750062	1.00E−05
P09874	Poly [ADP-ribose] polymerase 1	CVD2_195_PARP-1	O	1.78E−07	1	2.19E−11	3.81E−14	0.00192284	3.50E−16	0.210991816	1	2.04E−07	0.001644308
Q9UBX7	Kallikrein-11	ONC2_121_hK11	O	3.83E−05	1	1.20E−07	3.72E−09	0.001466544	2.15E−09	0.011603143	1	3.43E−06	0.1190456
Q96NY8	Nectin-4	ONC2_141_PVRL4	O	1.50E−06	1	1.57E−09	2.23E−07	0.096598347	2.39E−08	0.258520796	1	1.18E−07	0.242568821
Q8WXI7	Mucin-16	ONC2_191_MUC-16	O	9.15E−23	2.37E−08	4.49E−21	1.20E−16	0.001302092	1.45E−19	6.59E−18	0.752014614	9.16E−25	3.73E−11
P15328	Folate receptor alpha	ONC2_196_FR-alpha	O	3.91E−10	0.115430697	3.90E−12	2.50E−18	0.004482048	3.56E−22	0.000243851	1	5.22E−09	1.14E−05
P22223	Catenin beta-1	NEU_150_CDH3	O	3.91E−05	0.484466735	7.72E−05	1	0.055334855	1	0.118136808	1	0.010350682	1
Q16288	NT-3 growth factor receptor	NEU_190_NTRK3	O	0.030055578	1	0.039744561	2.31E−11	0.003961683	1.35E−12	1	1	1	0.015043749
P10145	Interleukin-8	INF_101_IL-8	O	8.17E−07	1	1.32E−09	9.27E−09	0.027613554	9.11E−10	0.027906542	1	3.41E−06	0.001205718
Q9P0M4	Interleukin-17C	INF_114_IL-17C	O	2.51E−05	0.043046188	0.000689734	1.07E−06	0.000623164	4.22E−06	1	1	1	0.044912595
P80511	Protein S100-A12	INF_175_EN-RAGE	O	4.87E−08	0.000153701	7.40E−05	4.64E−11	4.81E−08	2.15E−09	0.342001882	1	1	0.026901128

*NA* not applicable

**Table 3:** *p*-values for the individual proteins in the discovery and replication steps in the comparison of endometrial cancer to benign tumors, population controls and ovarian cancer

Uniprot no	Uniprot protein name	Proseek protein ID	Cancer	Endometrial cancer versus benign cysts		Endometrial cancer versus ovarian cancer 1–4		Endometrial cancer versus population controls	
				Discovery	Replication	Discovery	Replication	Discovery	Replication
Q16651	Prostasin	CVD2_173_PRSS8	OE	4.36E−09	6.88E−08	1	3.22E−07	0.01381015	0.928202706
P21741	Midkine	ONC2_161_MK	OE	3.65E−08	0.119795419	1	8.20E−09	0.026477007	1
Q14508	Major epididymis-specific protein E4 (WAP four-disulfide core domain protein 2)	ONC2_182_WFDC2	OE	6.28E−09	1.71E−10	1	2.83E−12	0.290677608	0.00019993
P22301	Interleukin 10	INF_162_IL-10	OE	4.23E−12	1	1	1	0.065101325	1
P35318	Adenomedulin	CVD2_103_ADM	E	2.48E−07	0.004662956	0.005277639	1	5.23E−05	0.608471686
P35475	Al pha-L-iduronidase	CVD2_116_IDUA	E	1.05E−05	0.954600936	6.83E−10	1	6.52E−08	0.443914788
P09237	Matrilysin	CVD2_167_MMP-7	E	7.21E−06	0.000194133	1	0.112528602	0.001380847	0.000174466
P15090	Fatty acid-binding protein, adipocyte	CVD3_129_FABP4	E	1.10E−06	1	0.01262521	1	0.000231174	0.023151067
Q8NBP7	Proprotein convertase subtilisin/kexin type 9	CVD3_161_PCSK9	E	1.43E−05	1	2.42E−05	1	0.002207816	1
Q01638	Interleukin-1 receptor-like 1	CVD3_176_ST2	E	1.35E−06	0.010291606	0.023539896	1	0.000249695	0.242686447
Q9UBR2	Cathepsin Z	CVD3_185_CTSZ	E	3.20E−07	1	3.59E−06	1	2.28E−06	1
015467	C–C motif chemokine 16	CVD3_196_CCL16	E	2.70E−06	1	0.48003849	0.006484386	0.005950521	1
Q9UBT3	Dickkopf-related protein 4	NEU_187_Dkk-4	E	2.60E−09	1	4.27E−05	0.252055749	0.000134061	0.022042978
P15692	Vascular endothelial growth factor A	INF_102_VEGF-A	E	0.00150827	1.88E−06	1	4.56E−05	0.156267791	1
P05231	Interleukin-6	INF_113_IL-6	E	0.000735048	1.41E−05	1	5.49E−06	1	1
P08581	Hepatocyte growth factor receptor	INF_156_HGF	E	6.46E−05	6.46E−05	6.46E−05	6.46E−05	6.46E−05	0.010198277

*NA* not applicable

**Fig. 3:**
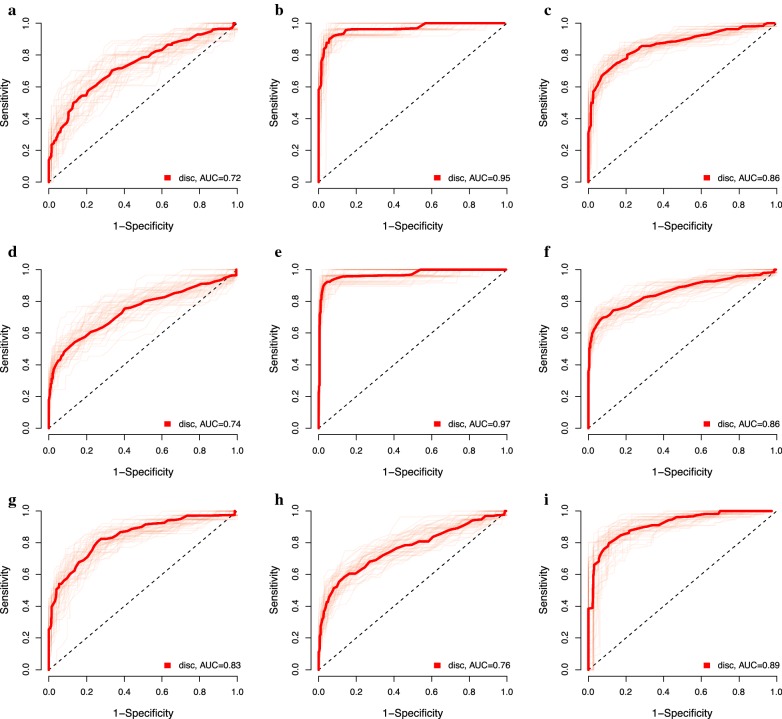
Reporter operator characteristic (ROC) for combinations of proteins in the discovery step. **a** Benign tumors versus Ovarian cancer stage I–II. **b** Benign tumors versus Ovarian cancer stage III–IV. **c** Benign tumors versus Ovarian cancer stage I–IV. **d** Population controls versus Ovarian cancer stage I–II. **e** Population controls versus Ovarian cancer stage III–IV. **f** Population controls versus Ovarian cancer stage I–IV. **g** Benign tumors versus Endometrial cancer. **h** Controls versus Endometrial cancer. **i** Ovarian cancer stage I-IV versus endometrial cancer versus

At the highest AUC (best point analysis) the 15-protein selected for OC could distinguish women with benign tumors from those with OC stage I–IV with a sensitivity = 0.79 and specificity = 0.85 (AUC = 0.86), from OC stage I–II with a sensitivity = 0.68 and specificity = 0.73 (AUC = 0.72) and from OC stage III–IV with a sensitivity = 0.93 and specificity = 0.93 (AUC = 0.95) (Table 4, Fig. 3). At a minimum sensitivity of 0.95, as a cutoff value for the biomarker panel to be used as a preoperative diagnostic test, the specificity to distinguish between benign ovarian tumors and OC stage III–IV was 0.54 (Table 4).

**Table 4:** Estimates of sensitivity and specificity for the protein panel based on the discovery and replication data

Comparison	Proteins	Best point				AUC	Minimum specificity of 0.95 (screening)				Minimum sensitivity of 0.95 (diagnostic)			
		Sensitivity		Specificity			Sensitivity		Specificity		Sensitivity		Specificity	
		Mean	SD	Mean	SD		Mean	SD	Mean	SD	Mean	SD	Mean	SD
*Analysis discovery*														
Benign tumors versus ovarian cancer stage I–II	All	0.68	0.07	0.73	0.08	0.72	0.33	0.11	0.96	0.01	0.96	0.01	0.07	0.08
Benign tumors versus ovarian cancer stage III–IV	All	0.93	0.04	0.93	0.03	0.95	0.86	0.07	0.96	0.01	0.96	0.00	0.54	0.25
Benign tumors versus ovarian cancer stage I–IV	All	0.79	0.04	0.85	0.04	0.86	0.61	0.07	0.96	0.01	0.96	0.01	0.18	0.10
Population ontrols versus ovarian cancer stage I–II	All	0.70	0.07	0.75	0.07	0.74	0.43	0.07	0.96	0.01	0.97	0.01	0.02	0.05
Population controls versus ovarian cancer stage III–IV	All	0.94	0.03	0.96	0.03	0.97	0.92	0.05	0.96	0.01	0.97	0.01	0.47	0.29
Population controls versus ovarian stage I–IV	All	0.75	0.05	0.87	0.05	0.86	0.66	0.06	0.95	0.00	0.96	0.01	0.13	0.10
Benign tumors versus endometrial cancer	All	0.77	0.05	0.79	0.05	0.83	0.45	0.11	0.96	0.01	0.97	0.01	0.09	0.11
Population controls versus endometrial cancer	All	0.69	0.06	0.78	0.06	0.76	0.43	0.07	0.95	0.00	0.97	0.01	0.07	0.07
Ovarian cancer versus endometrial cancer	All	0.84	0.04	0.88	0.04	0.89	0.64	0.17	0.97	0.01	0.96	0.01	0.42	0.16
*Replication*														
Benign tumors versus ovarian cancer stage I–II	All	0.73	0.08	0.74	0.07	0.76	0.29	0.11	0.97	0.01	0.97	0.01	0.19	0.14
Benign tumors versus ovarian cancer stage III–IV	All	0.88	0.03	0.92	0.03	0.92	0.81	0.07	0.96	0.01	0.96	0.01	0.58	0.15
Benign tumors versus ovarian cancer stage I–IV	All	0.83	0.04	0.87	0.04	0.89	0.68	0.10	0.97	0.01	0.96	0.01	0.48	0.12
Population ontrols versus ovarian cancer stage I–II	All	0.62	0.09	0.68	0.11	0.63	0.24	0.12	0.97	0.01	0.97	0.01	0.06	0.10
Population controls versus ovarian cancer stage III–IV	All	0.85	0.03	0.92	0.04	0.89	0.78	0.09	0.97	0.01	0.96	0.01	0.35	0.15
Population controls versus ovarian stage I–IV	All	0.77	0.05	0.85	0.05	0.86	0.62	0.10	0.96	0.01	0.96	0.01	0.21	0.11
Benign tumors versus endometrial cancer	All	0.70	0.06	0.67	0.07	0.72	0.22	0.09	0.97	0.01	0.96	0.01	0.25	0.08
Population controls versus endometrial cancer	All	0.64	0.07	0.72	0.08	0.71	0.24	0.14	0.97	0.01	0.96	0.01	0.10	0.06
Ovarian cancer versus endometrial cancer	All	0.73	0.03	0.77	0.04	0.78	0.36	0.06	0.96	0.00	0.96	0.00	0.11	0.06
Benign tumors versus ovarian cancer stage I–II	CA125/HE4	0.73	0.08	0.73	0.07	0.78	0.38	0.11	0.97	0.01	0.97	0.01	0.30	0.16
Benign tumors versus ovarian cancer stage III–IV	CA125/HE4	0.87	0.03	0.87	0.04	0.90	0.74	0.06	0.97	0.02	0.96	0.01	0.37	0.12
Benign tumors versus ovarian cancer stage I–IV	CA125/HE4	0.79	0.03	0.87	0.04	0.87	0.67	0.06	0.97	0.01	0.96	0.01	0.39	0.10
Population ontrols versus ovarian cancer stage I–II	CA125/HE4	0.59	0.08	0.77	0.10	0.66	0.35	0.11	0.97	0.01	0.97	0.01	0.14	0.07
Population controls versus ovarian cancer stage III–IV	CA125/HE4	0.84	0.03	0.89	0.04	0.86	0.74	0.07	0.97	0.01	0.96	0.00	0.17	0.09
Population controls versus ovarian stage I–IV	CA125/HE4	0.77	0.03	0.88	0.05	0.81	0.65	0.08	0.96	0.00	0.96	0.01	0.14	0.07

The 15-protein panel was also able to distinguish population controls from OC stage I–IV with a sensitivity = 0.75 and specificity = 0.87 (AUC = 0.86), from OC stage I–II with a sensitivity = 0.70 and specificity = 0.75 (AUC = 0.74) and from OC stage III–IV with a sensitivity = 0.94 and specificity = 0.96 (AUC = 0.97) (Table 4). At a specificity of 0.96, as a cutoff value for the biomarker panel to be useful in population screening, the sensitivity to distinguish controls from OC stage I–IV was 0.66, from OC stage I–II it was 0.43 and from OC stage III–IV it was 0.92 (Table 4).

**Fig. 4:**
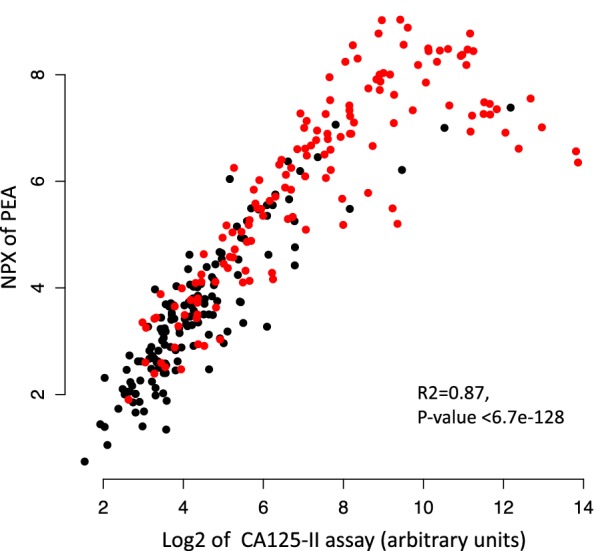
Correlation between CA125 values from the multiplex PEA used in the discovery step and from clinical ELISA, for benign tumors (in black) and ovarian cancer patients (in red)

Since plasma samples from benign tumors and OC stage I–IV had been analyzed previously for CA125 we compared our CA125 values from PEA with those from the Architect CA125-II assay (Abbott Diagnostics, Abbott Park, IL, USA). There was a strong correlation between the two measurements (*R*^2^ = 0.87, *p* value < 6.72e−128), except for the highest values which were outside the linear part of the range for PEA (Fig. 4, Additional file 2: Table 2).

For EC the 16-protein panel distinguished benign tumors from EC with a sensitivity = 0.77 and specificity = 0.79 (AUC = 0.83). OC stage I–IV could be distinguished from EC with a sensitivity of 0.84 and specificity of 0.88 (AUC = 0.89). Finally, the EC patients could be distinguished from population controls with a sensitivity = 0.69 and specificity = 0.78 (AUC = 0.76) (Table 4).

### Replication step

**Fig. 5:**
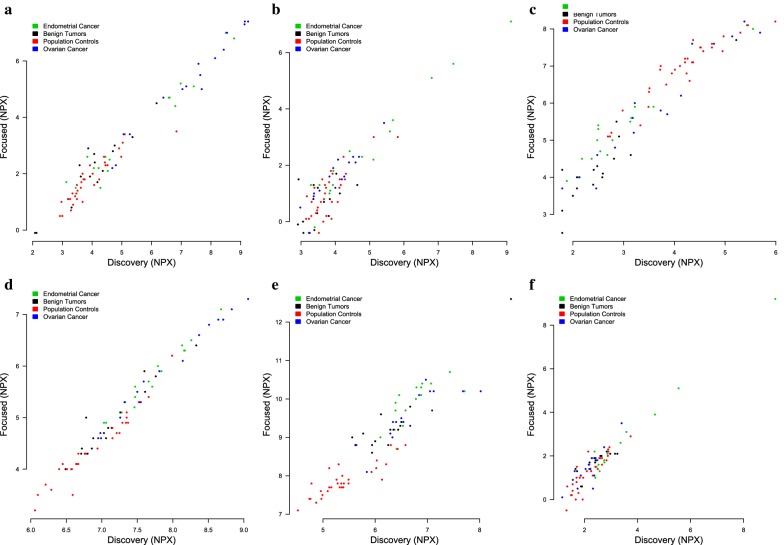
Correlation in protein abundance between the 92-plex panels and the focused protein panels for the six proteins **a** CA125, **b** IL10, **c** ENRAGE, **d** HE4, **e** MK, **f** Dkk4

Two focused multiplex PEA assays were developed for the 27 proteins identified in the discovery step, according to the protocol described by the manufacturer [15]. There was excellent correlation between the protein estimates from the multiplex assays used in the discovery step and the focused PEA assays used in the replication step (Fig. 5). Despite absolute differences in NPX-values (as expected since the dynamic range was shifted for some assays in the custom panel) the rank of different proteins was maintained (Fig. 5). Average intra- and interassay CV for 17 of the proteins included in the discovery and replication assays have previously been shown to be similar to the Olink’s commercial 92-plex screening panels [15]. Thus, the results of the PEA analyses used in the discovery and the replication steps were considered comparable.

**Fig. 6:**
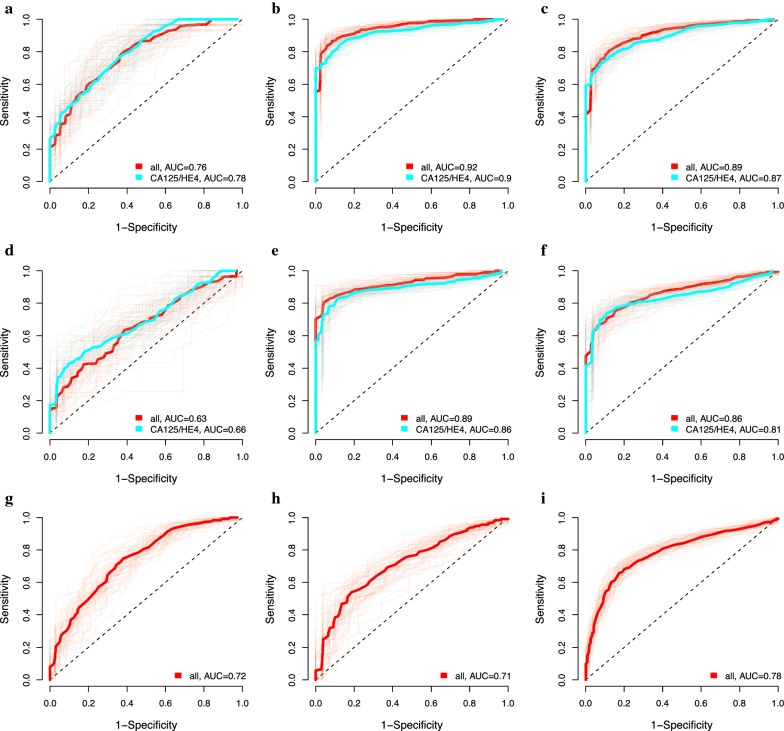
Reporter operator characteristic (ROC) for combinations of proteins in replication stage. The blue line is using CA125 and HE4 only. **a** Benign tumors versus Ovarian cancer stage I–II. **b** Benign tumors versus Ovarian cancer stage III–IV. **c** Benign tumors versus Ovarian cancer stage I–IV. **d** Population controls versus Ovarian cancer stage I–II. **e** Population controls versus Ovarian cancer stage III–IV. **f** Population controls versus Ovarian cancer stage I–IV. **g** Benign tumors versus Endometrial cancer. **h** Controls versus Endometrial cancer. **i** Ovarian cancer stage I–IV versus endometrial cancer versus

The focused PEA panels were then used to study an independent set of plasma samples from women with either of the two cancers, benign tumors and population controls (Table 1). For OC, the association was replicated for 13 of the 15 proteins and only IL-10 and CDH3 showed no association with OC. The protein panel distinguished benign tumors from OC stage I–IV with a sensitivity = 0.83 and specificity = 0.87 (AUC = 0.89), from OC stage I–II with a sensitivity = 0.73 and specificity = 0.74 (AUC = 0.76) and from OC stage III–IV with a sensitivity = 0.88 and specificity = 0.92 (AUC = 0.92) (Table 4, Fig. 6). The protein panel selected for OC had a significantly higher AUC than using only CA125 and HE4 for distinguishing benign tumors from OC (stage I–IV, *p *= 1.41e−13; stage 3–4, *p *= 9.56e−22). At a sensitivity of 0.96, the specificity to distinguish between benign ovarian tumors and OC stage I–IV was 0.48, stage I–II it was 0.19 and stage III–IV it was 0.58 (Table 4).

The selected protein panel distinguished population controls from OC stage I–IV with a sensitivity = 0.77 and specificity = 0.85 (AUC = 0.86), from OC stage I–II with a sensitivity = 0.62 and specificity = 0.68 (AUC = 0.63), and from OC stage III–IV with a sensitivity = 0.85 and specificity = 0.92 (AUC = 0.89) (Table 4, Fig. 6). At a minimum specificity of 0.95, the sensitivity to distinguish population controls from OC stage I–IV was 0.62, from OC stage I–II it was 0.24, and from OC stage III–IC it was 0.78 (Table 4).

**Fig. 7:**
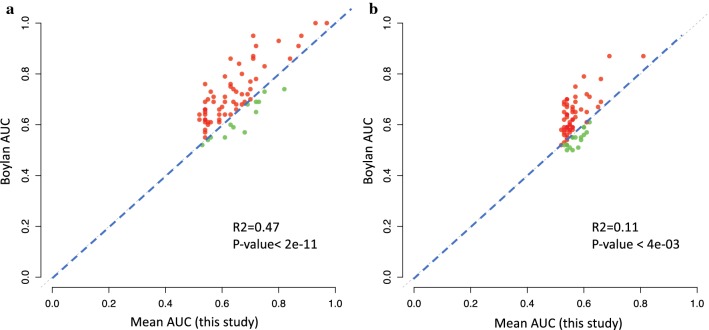
Correlation between AUC values for some of the proteins overlapping between our study and that by Boylan et al. [5] for the comparison between benign tumors and ovarian cancer stage III–IV (left panel) and I–II (right panel)

In a recent study, candidate biomarkers for OC were identified using the Olink Multiplex ONC I v2 panel [5]. That panel is discontinued by the manufacturer and replaced by the ONC II panel used here. However, some of the proteins overlap with the panels used here, and we therefore compared the AUC values for these proteins in the two studies (Fig. 7a). There was good correlation in the comparison of benign tumors to OC stage III–IV, but the AUC values were generally higher in the study by Boylan et al. [5]. By contrast, no correlation was found in the AUC values when comparing benign tumors and OC stage I–II (Fig. 7b).

Of the 16 proteins selected for EC, an association was replicated for nine (PRSS8, MK, WFDC2 (HE4), ADM, MMP-7, ST2, VEGF-A, IL-6, HGF), while seven proteins showed no association (IL-10, IDUA, FABP-4, PCSK9, CTSZ, CCL16, Dkk-4). The protein panel distinguished between benign tumors and EC with a sensitivity = 0.70 and a specificity = 0.67 (AUC = 0.72). Population controls could be distinguished from EC with a sensitivity = 0.64 and specificity = 0.72 (AUC = 0.71).

Of the 13 proteins that differed significantly between OC stage I–IV and EC in the discovery data, six (hK11, MUCIN-16, FR-alpha, NTRK3, EN-RAGE, HGF) showed a significant difference in the replication step (Tables 2, 3). OC stage I–IV could be distinguished from EC with a sensitivity = 0.73 and a specificity = 0.77 (AUC = 0.78).

## Discussion

We have searched for plasma proteins that could distinguish between patients with the gynecologic cancers OC and EC and benign tumors, using a two-step study design and a scalable technology for measuring protein abundance. The high degree of multiplexing of PEA panels enabled us to screen 441 unique proteins in search of suitable biomarker candidates. The ability to scale the PEA technology was then used to design two focused multiplex panels for the proteins selected in the discovery step. The abundance estimates for the proteins measured both in discovery step and the replication step showed high correlation and similar precision, testifying to that the PEA technology is scalable without compromising the performance for detection of individual proteins. In fact, by using a smaller Integrated Fluidic Circuit for the analysis, we were able to combine PEA assays for proteins with a greater difference in abundance than is possible using the 92-protein Integrated Fluidic Circuits.

### Proteins identified

Among the proteins we identified as biomarker candidates for OC some have been discussed previously [5], such as Mucin-16 (CA125) and Major epididymis-specific protein E4 (HE4), Midkine (MK) [16, 17], Kallikrein-11 (hK11) [18], Folate receptor alpha (FR) [19, 20] and Prostasin (PRSS8) [21, 22]. Boylan et al. [5] also using PEA technology further reported an association of OC with Interleukin-6 (IL-6) [23–25], Kallikrein-6 (KLK6) [26], Furin (FUR) [27], Chemokine (C-X-C motif) ligand 13 (CXCL13) and Tumor necrosis factor ligand superfamily member 14 (TNFSF14), but none of these proteins were among our top candidates. A number of the proteins we selected for the replication step were not studied by Boylan et al. [5] such as Interleukin-8 (IL-8), Nectin-4 (PVRL4), Interleukin-17C (IL17C), Poly (ADP-ribose) polymerase-1 (PARP-1), Superoxide dismutase (Mn), mitochondrial (SOD2) and Protein S100-A12 (EN-RAGE). Several of these have previously been noted in connection to OC. The level of Interleukin-8 has been proposed as a diagnostic and prognostic biomarker for OC [28–30]. Nectin-4 is overexpressed in epithelial cancers including OC and has been proposed as a therapeutic target [31]. Interleukin-17C has been shown to be tumor-promoting in OC cell models [32]. Poly (ADP-ribose) polymerase 1 is overexpressed in OC and may enhance angiogenesis by upregulating Vascular endothelial growth factor A (VEGF-A) [33]. Genetic variation in Neurotrophic tyrosine receptor kinase 3 (NTRK3) has been associated with prognosis of OC and suggested to predict platinum resistance in OC patients [34]. Superoxide dismutase (Mn), mitochondrial, is highly expressed in OC and has been shown to increase tumor development and metastatic spread [35].

Among the nine proteins (Prostasin (PRSS8), Midkine (Mk), Major epididymis-specific protein E4 (HE4), Adenomedulin (ADM), Matrilysin (MMP-7), Interleukin-1 receptor-like 1 (ST2), Vascular endothelial growth factor A (VEGF-A), Interleukin-6 (IL-6), Hepatocyte growth factor receptor (HGF)) associated with EC in the replication step, several have been discussed in relation to EC. Midkine has been proposed as a serum biomarker for high-risk EC patients, and preoperative serum levels have been shown to correlate with lymph node metastasis [36]. Adrenomedullin expression is upregulated in post-menopausal endometria, and is increased during progression from benign endometrium to type-1 adenocarcinoma [37, 38]. Fatty acid-binding protein (FABP4) has been proposed as a diagnostic biomarker for EC [39, 40] and showed a significant association in our discovery step analysis, but not in the replication step.

### Preoperative diagnostic

Our protein panel has a sensitivity = 0.83 and specificity = 0.87 to distinguish between benign tumors and OC stage I–IV. The specificity of our protein panel is somewhat lower than the 0.91 of Overa^®^, while the sensitivity is higher than the 0.69 reported for Overa^®^ [4]. Focusing on OC stage III–IV we have a sensitivity = 0.88 and specificity = 0.92, which is significantly higher than using only CA125 and HE4.

A test for triaging women with adnexal ovarian mass should have better performance than TVU. A recent study showed that TVU in the hands of specially trained gynecologic sonographers can achieve an AUC = 0.92 [41]. However, the performance of ordinary gynecologists is generally lower. For instance, among women with a TVU indication of adnexal ovarian mass that are diagnosed by surgical sampling, 58% have been reported to have benign tumors, 30% have OC stage I–IV and the remaining 15% borderline tumors [42, 43]. Among the 30% of women with OC, 15% have OC stage III–IV. Based on these estimates, clinical diagnosis by surgical sampling has a specificity for OC stage I–IV of 0.30 and for OC III–IV of 0.15. At a minimum sensitivity of 0.96, used as a threshold for a preoperative diagnostic test, our protein panel can distinguish between benign tumors and OC stage III–IV with a specificity of 0.58. This indicates that the number of women with OC stage III–IV among those stratified for surgical sampling based on TVU could be increased from 15% when using TVU to 58% by using the protein panel. For OC stage I–IV, the protein panel show a specificity of 0.48, while the specificity of TVU is 0.30. This correspond to a 50% increase of the specificity. The combined use of both TVU and the biomarker test is likely to give even higher specificity.

Boylan et al. [5] used the ONC Iv2 panel to search for candidate biomarkers for OC. Several of the proteins found to be associated with OC in their study were also on our list of candidates, such as Major epididymis-specific protein E4, Midkine, Kallikrein-11, Folate receptor alpha. Interleukin-6, and Prostasin. Their list of top proteins had consistently higher AUC values than our estimates (Fig. 7). This may reflect differences in design between the studies. Boylan et al. [5] used a single set of cases and controls for identification of proteins, while we used a two-step analysis with two independent set of clinical materials, and in the replication step we also included OC patients from two different hospitals. Our study thus includes several factors that could introduce variation, such as multiple patient cohorts from both the same and different hospitals, different PEA analysis rounds and using both 92-plex and focused PEA panels, and finally the use of a replication step to verify initial findings. Together these factors are likely to reduce the overall performance characteristics of the assay, while at the same time result in more realistic performance indicators.

We also identified a set of protein biomarkers that can be used to distinguish between benign tumors and EC. Biomarkers have previously been described for EC, and good diagnostic accuracy has been reported for e.g. Major epididymis-specific protein E4 (HE4), Growth/differentiation factor 15 (GDF-15), C-Jun-amino-terminal kinase-interacting protein 4 (JIP-4), JNK-interacting protein 4 (SPAG9), Chitinase-3-like protein 1 (YKL-40), Interleukin-31 (IL-31) and Interleukin-33 (IL-33) [11]. Further studies are needed to compare the performance of these candidates with the ones identified in the present study.

### Population screening

Our results indicate that the focused protein panel at a specificity of 0.96 has a high sensitivity to distinguish population controls from women with OC stage I–IV and stage III–IV, while lower for stage I–II. To determine the potential of using the protein panel in population screening, further studies are needed of based on samples collected at distinct time-points prior to diagnosis. A recent evaluation of four markers (CA125, HE4, CA72.4, and CA15.3) for OC showed that the performance declined with increasing time between sample collection and time of diagnosis [44]. Serial sampling could enable the use of individual baseline values, and testing women at 3 months rather than 6-12-month intervals using ROCA has been shown to result in better sensitivity and high specificity for detection of early-stage disease [45].

In summary, we have used a two-step strategy to identify plasma proteins that can be used to distinguish benign tumors from OC or EC and for differential diagnostic procedures of women with suspicious pelvic mass. The biomarker panels could be useful in population screening and to identify women in need of TVU examination at a specialized gynecologic ultrasound unit.

## Additional files

**Additional file 1: Table 1.** P-values of individual proteins in the discovery step.

**Additional file 2: Table 2.** CA125 values in the clinical analyses and the PEA NPX values for some of the benign and ovarian cancer cohorts used in the discovery analysis.
