# Supplementary material for: A two-step strategy for identification of plasma protein biomarkers for endometrial and ovarian cancer
Source: Clin Proteomics. 2018 Dec 1;15:38. doi: 10.1186/s12014-018-9216-y (PMC6271635; doi:10.1186/s12014-018-9216-y)
Supplement: Supplementary file 1 — Additional file 1: Table 1. P-values of individual proteins in the discovery step. [file 12014_2018_9216_MOESM1_ESM.pdf]

Supplementary Table 1

Selected för cancer (E, O)	OLINK Panel and Protein ID	Mean NPX in benign cysts	Sample size benign	Mean NPX Ovarian cancer stage 1-4	Sample size Ovarian cancer 1-4	P-value benign vs Ovarian cancer stage 1-4	Mean NPX Ovarian cancer stage 1-2	Sample size Ovarian cancer 1-2	P-value benign vs Ovarian cancer stage 1-2	Mean NPX Ovarian cancer stage 3-4	Sample size Ovarian cancer 3-4	P-value benign vs Ovarian cancer stage 3-4	Mean NPX Endometrial cancer	Sample size Endometrial cancer	P-value benign vs Endometrial cancer	P-value Endometrial cancer vs Ovarian cancer 1-4	Mean NPX Controls	Sample size Controls	P-value Ovarian cancer 1-4 vs controls	P-value Ovarian cancer 1-2 vs controls	P-value Ovarian cancer 3-4 vs controls	P-value Controls vs Endometrial cancer
	CVD2_101_BMP-6	3,481645945	150	3,363073618	106	1	3,271127426	54	1	3,458556201	52	1	3,630445032	73	1	0,007177775	3,480613687	398	1	1	1	1
	CVD2_102_ANG-1	7,725651904	150	7,693844357	106	1	7,459644497	54	1	7,937051904	52	1	8,220134096	73	0,393824461	0,539150401	7,801406743	399	1	1	1	0,001272417
E	CVD2_103_ADM	5,96631587	150	6,136372474	106	1	5,989723278	54	1	6,288662024	52	0,216480273	6,541904911	73	2,48E-07	0,005277639	6,120165333	399	1	1	1	5,23E-05
	CVD2_105_CD40-L	4,994121445	150	4,869760438	106	1	4,778743667	54	1	4,964277855	52	1	5,298142449	73	1	1	5,010258004	399	1	1	1	1
	CVD2_106_SLAMF7	1,403369424	116	1,393601982	86	1	1,462637717	41	1	1,330702757	45	1	1,486917811	62	1	1	1,422127813	362	1	1	1	1
	CVD2_107_PlGF	6,67909257	150	6,687828419	106	1	6,569907385	54	1	6,810284878	52	1	6,965709008	73	0,001197694	0,005834657	6,731060312	399	1	1	1	0,000106054
	CVD2_108_ADAM-TS13	5,195459968	150	5,068783239	106	0,052476306	5,10954145	54	1	5,026457404	52	0,113062707	5,270411567	73	1	6,82E-06	5,173732011	399	0,00272141	1	0,039596633	0,168452701
	CVD2_109_Protein-BOC	4,241406225	150	4,25505654	106	1	4,16719141	54	1	4,346301097	52	1	4,311983394	73	1	1	4,264748161	399	1	1	1	1
	CVD2_110_IL-4RA	2,303500548	150	2,622111869	106	0,104116811	2,44378203	54	1	2,807300548	52	0,001812214	2,609971781	73	0,269584268	1	2,445714527	398	1	1	0,031401837	1
	CVD2_111_SRC	5,937420564	150	5,973155155	106	1	6,086509452	54	1	5,855441076	52	1	5,685246134	73	1	1	5,88646465	399	0,147332891	0,806602943	1	1
	CVD2_112_IL-1ra	3,67575898	150	3,925460867	106	1	3,806047869	54	1	4,049466673	52	0,586258041	4,294830213	73	4,68E-05	1	3,893368658	398	1	1	1	0,084316717
	CVD2_113_IL-6	2,196337375	150	2,989610331	106	0,016651244	2,687307745	54	1	3,303539939	52	0,000964597	3,242393996	73	3,25E-05	1	2,647367483	398	1	1	1	1
	CVD2_114_TNFRSF10A	2,441212895	150	2,539737423	106	1	2,367183265	54	1	2,71892828	52	0,722769254	2,791675909	73	0,000127167	0,149252753	2,525925798	398	1	1	1	0,002957607
	CVD2_115_STK4	2,449459285	149	2,483943785	105	1	2,470070769	54	1	2,498632861	51	1	2,092848547	72	1	1	2,375212677	392	1	1	1	1
E	CVD2_116_IDUA	4,095379339	150	3,891456069	106	1	3,887364525	54	1	3,895704981	52	1	4,523046919	73	1,05E-05	6,83E-10	4,122582565	399	0,055020173	1	1	6,52E-08
	CVD2_117_TNFRSF11A	4,512842699	150	4,64552446	106	1	4,486146403	54	1	4,811032443	52	0,499819642	4,911712106	73	0,001217978	0,825191519	4,632469581	399	1	1	1	0,019820132
	CVD2_118_PAR-1	6,612894082	150	6,596614837	106	1	6,510479267	54	1	6,686063313	52	1	6,777965315	73	1	1	6,62604677	399	1	1	1	0,977477479
	CVD2_120_TRAIL-R2	4,479053553	150	4,707906383	106	1	4,499712812	54	1	4,924107399	52	0,045481666	4,867302868	73	0,000157983	1	4,632604091	399	1	1	1	0,043927866
	CVD2_121_PRSS27	7,363753994	150	7,231437013	106	1	7,184857698	54	1	7,27980784	52	1	7,462044405	73	1	0,716887698	7,355750768	399	1	1	1	1
	CVD2_122_TIE2	7,188880103	150	7,179615953	106	1	7,162991215	54	1	7,196880103	52	1	7,391400651	73	0,008189389	0,009222929	7,232794082	399	1	1	1	0,111777214
	CVD2_123_TF	4,856973417	150	4,680991027	106	1	4,689432677	54	1	4,672224699	52	1	5,092662002	73	0,01942701	7,62E-07	4,828936858	399	1	1	1	0,000137385
	CVD2_124_IL1RL2	4,054025993	150	3,98029266	106	1	3,951774141	54	1	4,009908044	52	1	4,460018687	73	0,000312118	1,42E-06	4,121717391	398	1	1	1	0,001560567
	CVD2_125_PDGF-subunit-B	8,204693391	150	8,130342447	106	1	7,952456354	54	1	8,315070314	52	1	8,672849555	73	1	0,695943049	8,266133176	399	1	1	1	0,009401956
	CVD2_126_IL27	3,128222924	150	3,235571352	106	1	3,180452554	54	1	3,292810104	52	1	3,396074066	73	0,001236423	1	3,200204645	399	1	1	1	0,00427901
	CVD2_127_IL-17D	2,009449343	150	1,850090853	106	1	1,881338232	54	1	1,817641651	52	1	2,209024686	73	0,225803549	1,19E-05	1,989384827	398	1	1	1	0,003556277
	CVD2_128_CXCL1	7,497458824	150	7,613180837	106	1	7,480029195	54	1	7,751453696	52	1	7,510183939	73	1	1	7,519927642	399	1	1	1	1
	CVD2_129_LOX-1	6,585114375	150	6,709270349	106	1	6,708721782	54	1	6,709840016	52	1	6,95752442	73	0,009325703	1	6,687282117	399	1	1	1	1
	CVD2_130_Gal-9	6,054205235	150	6,172451776	106	1	6,033968198	54	1	6,316261645	52	0,122689856	6,321941308	73	0,012642637	1	6,132591256	399	1	1	1	0,297822118
	CVD2_131_GIF	5,077061003	150	4,697489934	106	1	4,787046188	54	1	4,604489208	52	1	5,402089314	73	1	0,00064527	5,015006165	399	0,206167322	1	1	0,832745916
	CVD2_132_SCF	8,777710369	150	8,469384583	106	0,288630327	8,674880739	54	1	8,255984728	52	0,002925463	8,9395332	73	1	0,001121805	8,701203917	399	1	1	0,014175222	0,005517437
	CVD2_133_IL-18	7,726970046	150	7,718982625	106	1	7,625303379	54	1	7,816264918	52	1	7,940029407	73	1	1	7,775410906	399	1	1	1	1
	CVD2_134_FGF-21	5,33241564	150	5,794208093	106	1	5,591860084	54	1	6,004338717	52	1	5,932963585	73	1	1	5,601152199	398	1	1	1	1
	CVD2_135_PIgR	5,646753654	150	5,625563717	106	1	5,635361062	54	1	5,615389552	52	1	5,729620321	73	1	1	5,651582687	399	1	1	1	1
	CVD2_136_RAGE	4,387003464	150	4,328572018	106	1	4,333047909	54	1	4,323923977	52	1	4,522050953	73	1	1	4,395619593	399	1	1	1	1
O	CVD2_137_SOD2	7,814038649	150	8,020385819	106	0,00066448	7,95394976	54	1	8,089377111	52	0,000688683	7,925235909	73	1	1	7,9039408	399	1	1	0,25750062	1
	CVD2_138_CTRC	9,528131154	150	9,319401595	106	1	9,283168192	54	1	9,35702859	52	1	9,842660835	73	1	0,107307133	9,531588144	399	1	1	1	0,342541728
	CVD2_139_FGF-23	3,286537199	150	3,429698206	106	1	3,276270533	54	1	3,589026943	52	1	3,794946332	73	0,0181745	1	3,44403935	398	1	1	1	0,355707089
	CVD2_140_SPON2	8,477261363	150	8,540665137	106	1	8,478135437	54	1	8,605599825	52	0,960131581	8,605636706	73	0,227493254	1	8,520136632	399	1	1	1	1
	CVD2_141_GH	8,347841386	150	8,710994845	106	1	8,646700645	54	1	8,777761899	52	1	7,412738189	73	0,25157347	0,001277927	8,278247837	399	1	1	1	0,705579184
	CVD2_142_FS	9,769010809	150	9,925902633	106	1	9,816136735	54	1	10,0398903	52	1	10,12785921	73	0,000917278	1	9,882683928	399	1	1	1	0,348392367
	CVD2_143_GLO1	6,335979788	150	5,846080417	106	0,163549561	5,788498307	54	0,52189638	5,905877224	52	1	6,304728647	73	1	1	6,157614197	399	0,073592846	0,354932724	1	1
	CVD2_144_CD84	4,663722136	150	4,627542262	106	1	4,563062877	54	1	4,694501623	52	1	4,901888346	73	0,26626366	0,040590933	4,696838265	399	1	1	1	0,16804812
	CVD2_145_PAPPA	1,443060517	150	1,339747309	106	1	1,352490146	54	1	1,326514363	52	1	1,763887914	73	0,001849665	5,36E-05	1,474849764	107	5,74E-07	6,30E-05	0,001723764	1,68E-14
	CVD2_148_SERPINA12	3,306777391	145	3,133247266	91	1	3,173467046	48	1	3,088350767	43	1	3,198537468	71	1	1	3,218784965	373	1	1	1	1
	CVD2_149_REN	6,48724371	150	6,650588364	106	1	6,569480747	54	1	6,734815505	52	1	6,819270194	73	1	1	6,612255538	399	1	1	1	1
	CVD2_150_DECR1	4,005260793	150	4,176191611	106	1	4,078371904	54	1	4,277773614	52	1	4,125913761	73	1	1	4,064330686	399	1	1	1	1
	CVD2_151_MERTK	3,269337653	150	3,209337716	106	1	3,219700616	54	1	3,198596628	52	1	3,583815279	73	9,74E-05	8,81E-07	3,301872062	398	1	1	1	3,61E-05
	CVD2_152_TIM	8,235455979	150	8,467657237	106	1	8,442863386	54	1	8,493404697	52	1	8,661186572	73	0,904840349	1	8,311423721	399	1	1	1	1
	CVD2_153_THBS2	5,039461561	150	5,120016278	106	1	5,065372672	54	1	5,176761561	52	1	5,207001287	73	0,025666953	1	5,100901346	399	1	1	1	1
	CVD2_154_TM	7,617059055	150	7,545428866	106	1	7,449370166	54	1	7,645182132	52	1	7,912300151	73	0,002594253	8,70E-06	7,646565507	399	1	0,336812436	1	4,89E-05
	CVD2_155_VSIG2	2,950802056	150	2,886501427	106	1	2,824453908	54	1	2,950935389	52	1	3,204976485	73	1	0,379941349	2,972682701	398	1	1	1	1
	CVD2_156_AMBP	6,243520774	150	6,253462912	106	1	6,213054107	54	1	6,295425902	52	1	6,440017577	73	0,009740505	0,104133834	6,279640129	399	1	1	1	0,000526313
	CVD2_157_PRELP	5,668240169	150	5,602105578	106	1	5,578010539	54	1	5,627127348	52	1	5,7820009	73	0,907704886	0,005527061	5,658312212	399	1	1	1	0,017130011
	CVD2_158_HO-1	10,224577	150	10,12272543	106	1	10,17093996	54	1	10,07265649	52	1	10,46548841	73	1	0,00864432	10,23313829	399	1	1	1	1
	CVD2_159_XCL1	4,346157098	150	4,252777224	106	1	4,212186728	54	1	4,294928893	52	1	4,481643856	73	1	1	4,332906561	399	1	1	1	1
	CVD2_160_IL16	4,521418106	150	4,4306332	106	1	4,470699587	54	1	4,389025798	52	1	4,778327695	73	1	0,015616253	4,538438536	399	1	1	1	1
	CVD2_161_SORT1	5,171982408	150	5,194784923	106	1	5,107419445	54	1	5,285510613	52	1	5,532665513	73	0,000554629	0,006580241	5,235006064	399	1	1	1	4,32E-09
	CVD2_162_CEACAM8	3,566353561	150	3,641987523	106	1	3,651998005	54	1	3,631592022	52	1	3,858169999	73	1	1	3,660913776	398	1	1	1	1
	CVD2_163_PTX3	1,982613408	150	2,244470012	106	0,339629674	2,112080075	54	1	2,38195187	52	0,007829406	2,17370108	73	1	1	2,087187602	396	1	1	0,065486207	1
	CVD2_164_PSGL-1	4,07035876	150	3,90189964	106	0,025782246	3,977951352	54	1	3,822922862	52	0,005647133	4,122459216	73	1	0,005026316	4,022348007	399	0,023410238	1	0,004178196	1
	CVD2_165_CCL17	6,553583827	150	6,550372506	106	1	6,39351716	54	1	6,71326075	52	1	7,077307114	73	1	1	6,65289888	399	1	1	1	1
	CVD2_166_CCL3	1,262836027	150	1,581390744	106	0,112103715	1,443784175	54	1	1,724289873	52	0,009427705	1,507074383	73	0,176684327	1	1,437536297	383	1	1	1	1
E	CVD2_167_MMP-7	8,865116355	150	9,186555349	106	1	8,854242281	54	1	9,531649689	52	0,001923533	9,491170237	73	7,21E-06	1	9,084929259	399	1	1	0,017102476	0,001380847
	CVD2_168_IgG-Fc-receptor-II-b	1,102211692	143	1,120398995	102	1	1,127798181	53	1	1,112395793	49	1	1,134532981	72	1	1	1,098566562	341	1	1	1	1
	CVD2_169_ITGB1BP2	2,730875417	141	2,784744453	103	1	2,674315803	53	1	2,901798822	50	1	2,345187057	68	1	1	2,65758749	263	1	1	1	1
	CVD2_170_DCN	4,440043726	150	4,382618569	106	1	4,322384467	54	1	4,445169367	52	1	4,621209936	73	0,085389274	0,00199539	4,447692113	399	1	1	1	0,003336462
	CVD2_171_Dkk-1	7,839750614	150	7,8603003	106	1	7,714832096	54	1	8,011363435	52	1	8,224400843	73	0,20118055	0,615923332	7,896173195	399	1	1	1	0,067054658
	CVD2_172_LPL	8,817156654	150	8,551066088	106	0,016758902	8,586964061	54	1	8,513787423	52	0,027074995	9,017844325	73	1	1,71E-05	8,771734073	399	0,004973421	1	0,016766775	0,09205858
OE	CVD2_173_PRSS8	7,894378005	150	8,398240899	106	1,08E-08	8,17262245	54	0,437395062	8,63253698	52	9,05E-11	8,389707686	73	4,36E-09	1	8,147398436	399	0,028270178	1	1,29E-06	0,01381015
	CVD2_174_AGRP	2,308358383	150	2,340476622	106	1	2,303002828	54	1	2,379391717	52	1	2,057439662	73	1	0,53072755	2,257053007	398	1	1	1	1
	CVD2_175_HB-EGF	5,087907009	150	5,043002607	106	1	4,922188491	54	1	5,16846342	52	1	5,331643083	73	0,679161776	0,026205818	5,10258873	399	1	0,027969081	1	1
	CVD2_176_GDF-2	3,678160847	150	3,338547011	106	0,034715073	3,504427514	54	1	3,166286488	52	0,008863927	3,788011989	73	1	0,00273633	3,585287729	398	0,888252611	1	0,030670969	0,03923647
	CVD2_177_FABP2	8,075863283	150	7,815541271	106	1	7,796470691	54	1	7,835345334	52	1	8,134565566	73	1	1	8,01322995	399	1	1	1	1
	CVD2_178_THPO	1,551593095	150	1,476933975	106	1	1,468170872	54	1	1,48603412	52	1	1,794068894	73	0,001680055	0,000100444	1,569640406	398	1	1	1	0,002510029
	CVD2_179_MARCO	5,472919339	150	5,477826257	106	1	5,433867487	54	1	5,523475749	52	1	5,639600617	73	0,004771722	0,047210207	5,50964407	399	1	1	1	0,031412593
	CVD2_180_GT	0,961441389	136	0,815731127	94	1	0,787114105	47	1	0,844348148	47	1	0,881840549	70	1	1	0,883681772	320	1	1	1	1
	CVD2_181_BNP	1,264629978	104	1,080695589	85	1	0,916379063	42	1	1,241190801	43	1	1,289513333	61	1	1	1,176580121	147	1	1	1	1
	CVD2_182_MMP-12	6,358160829	150	6,694187245	106	1	6,514257126	54	1	6,881037753	52	1	6,707207405	73	1	1	6,52656298	399	1	1	1	1
	CVD2_183_ACE2	2,649362792	150	2,784825686	106	1	2,676614644	54	1	2,89719869	52	1	3,0660185	73	0,122053531	1	2,767666018	398	1	1	1	1
	CVD2_184_PD-L2	1,773978797	150	1,762046722	106	1	1,687438056	54	1	1,839524951	52	1	1,985507564	73	0,046459853	0,044466725	1,811243313	398	1	1	1	0,369169398
	CVD2_185_CTSL1	4,503569925	150	4,733473069	106	0,139617891	4,561488443	54	1	4,912072489	52	0,000333435	4,781719925	72	0,033286255	1	4,607017544	399	1	1	0,026736034	1
	CVD2_186_hOSCAR	9,079564237	150	9,081565495	106	1	9,056771645	54	1	9,107312955	52	1	9,22920442	73	0,141667276	0,272323216	9,106487893	399	1	1	1	0,348210208
	CVD2_187_TNFRSF13B	7,071479869	150	7,018385529	106	1	7,030146536	54	1	7,006172177	52	1	7,201288088	73	1	1	7,08396374	399	1	1	1	1
	CVD2_188_TGM2	5,758557862	150	5,696738994	106	1	5,656054159	54	1	5,738988632	52	1	5,27078526	73	0,002678464	0,315371792	5,615618077	399	1	1	1	0,005589205
	CVD2_189_LEP	5,792425498	150	5,239978957	106	0,02806681	5,482906979	54	1	4,987707549	52	0,04897395	6,331379836	73	0,395410923	7,37E-07	5,739251194	399	0,003477248	1	0,020106039	0,134133603
	CVD2_190_CA5A	1,622148536	116	1,735726607	89	1	1,413094896	45	1	2,065690856	44	1	2,264019733	68	1	1	1,787189826	320	1	1	1	1
	CVD2_191_HSP-27	9,788212984	150	9,892141286	106	1	9,884835206	54	1	9,899728369	52	1	9,931791066	73	1	1	9,843903307	399	1	1	1	1
	CVD2_192_CD4	3,562159496	150	3,546475219	106	1	3,478589125	54	1	3,616972316	52	1	3,666790546	73	1	1	3,568045517	399	1	1	1	1
	CVD2_193_NEMO	4,984288742	150	4,90505478	106	1	4,834673927	54	1	4,978142588	52	1	4,784488742	73	1	1	4,908117774	399	1	1	1	1
	CVD2_194_VEGF-D	6,171408909	150	6,014869287	106	1	6,011060761	54	1	6,018824294	52	1	6,148044526	73	1	1	6,107219662	399	1	1	1	1
O	CVD2_195_PARP-1	1,364571381	150	2,243685847	106	1,78E-07	1,767815826	54	1	2,737858561	52	2,19E-11	1,789238961	73	0,148595394	1	1,682833747	130	0,210991816	1	2,04E-07	1
	CVD2_196_HAOX1	3,348528397	150	3,580254184	106	1	3,564202471	54	1	3,596923269	52	1	4,421132964	73	0,018181065	1	3,664402591	398	1	1	1	1
	CVD3_101_TNFRSF14	3,881186568	148	3,936080391	105	1	3,839572454	54	1	4,038265265	51	1	4,141929811	74	0,019056888	0,510680462	3,963091974	397	1	1	1	0,051193909
	CVD3_102_LDL-receptor	3,540326673	148	3,409369144	105	1	3,478009356	54	1	3,336691273	51	1	3,843029376	74	0,216980136	0,001867922	3,588407754	397	1	1	1	1
	CVD3_103_ITGB2	4,128984002	148	4,048442174	105	1	4,079114132	54	1	4,015965984	51	1	4,181821839	74	1	1	4,117065083	397	1	1	1	1
	CVD3_105_IL-17RA	3,302856423	148	3,260878302	105	1	3,286084651	54	1	3,234189227	51	1	3,481234802	74	1	0,968308623	3,350072639	397	1	1	1	1
	CVD3_106_TNF-R2	3,794132004	148	3,94268284	105	1	3,812825697	54	1	4,080178638	51	0,54649971	4,133861733	74	0,000683924	1	3,928186058	397	1	1	1	0,060043045
	CVD3_107_MMP-9	3,146247975	148	3,104876675	105	1	3,1892735	54	1	3,01551533	51	1	3,603342569	74	0,18517478	0,451092792	3,244856083	397	1	1	1	1
	CVD3_108_EPHB4	1,339077147	148	1,393044972	105	1	1,310473543	54	1	1,480473543	51	1	1,54205012	74	0,008342653	0,613893654	1,415509579	395	1	1	1	0,360650201
	CVD3_109_IL2-RA	3,25319324	148	3,527771103	105	0,164833978	3,252988035	54	1	3,818717882	51	0,00029073	3,531706753	74	0,040965555	1	3,418382429	397	1	1	0,022375478	1
	CVD3_110_OPG	2,428319865	148	2,52807083	105	1	2,427261307	54	1	2,634810326	51	1	2,714873919	74	0,021381501	1	2,516009054	397	1	1	1	0,190733504
	CVD3_111_ALCAM	4,238222513	148	4,205212861	105	1	4,175382173	54	1	4,236798295	51	1	4,446127919	74	0,001332938	0,000388428	4,280371162	397	1	1	1	0,005033804
	CVD3_112_TFF3	4,60258217	148	4,749717949	105	1	4,770437526	54	1	4,727779574	51	1	5,140757846	74	0,006270755	0,886212062	4,802865954	397	1	1	1	0,10329711
	CVD3_113_SELP	8,242861223	148	8,306652728	105	1	8,262991353	54	1	8,35288242	51	1	8,489347709	74	1	1	8,312293655	397	1	1	1	1
	CVD3_114_CSTB	4,000324393	148	4,076915126	105	1	3,901745814	54	1	4,262388516	51	1	4,324783852	74	0,110010128	1	4,085973041	396	1	1	1	0,899718637
	CVD3_115_MCP-1	2,359294846	148	2,435170651	105	1	2,31021827	54	1	2,567473172	51	0,909222979	2,689227279	74	0,057649144	1	2,469848901	397	1	0,235485629	1	1
	CVD3_116_CD163	6,67609068	148	6,789750268	105	1	6,682834924	54	1	6,90295475	51	1	7,015347437	74	0,003920042	1	6,79005014	397	1	1	1	0,221724437
	CVD3_117_Gal-3	4,379243754	148	4,367117628	105	1	4,295154665	54	1	4,443313706	51	1	4,602081592	74	0,112379742	0,228759133	4,425757267	397	1	1	1	0,391987026
	CVD3_118_GRN	2,868248792	148	2,894275175	105	1	2,825888932	54	1	2,966684139	51	1	3,143451494	74	0,000344438	0,002645605	2,951181224	397	1	1	1	0,004369361
	CVD3_120_MEPE	2,354826945	148	2,170931192	105	1	2,237169288	54	1	2,100796739	51	0,698280206	2,590637756	74	0,490196479	5,07E-06	2,369610729	397	1	1	1	0,072190653
	CVD3_121_BLM-hydrolase	5,154369696	65	4,965985081	50	1	4,882604129	21	1	5,026364391	29	1	5,072353502	38	1	1	5,087043905	138	1	1	1	1
	CVD3_122_PLC	5,945989924	148	5,891113477	105	1	5,84660554	54	1	5,938239527	51	1	6,213016951	74	0,016422814	0,001790054	5,995152087	397	1	1	1	0,025043273
	CVD3_123_LTBR	2,700538907	148	2,761483566	105	1	2,66345182	54	1	2,865281885	51	1	2,975538907	74	0,001461724	0,326854623	2,786592961	397	1	1	1	0,004699969
	CVD3_124_Notch-3	2,990778031	148	2,874670567	105	1	2,848908662	54	1	2,901947877	51	1	3,313007761	74	0,001341475	4,99E-06	3,03365641	397	1	1	1	0,000241367
	CVD3_125_TIMP4	4,130792566	148	4,076696684	105	1	4,136405679	54	1	4,013475396	51	1	4,543292566	74	5,31E-05	9,07E-06	4,202427701	397	1	1	1	4,60E-05
	CVD3_126_CNTN1	2,305447444	148	2,075887598	105	0,686990231	2,174411408	54	1	1,97156827	51	0,174060327	2,405852849	74	1	0,048540826	2,280177173	397	1	1	0,202074921	1
	CVD3_127_CDH5	2,942596603	148	2,798211146	105	1	2,823184691	54	1	2,771768569	51	1	3,189691197	74	0,008181667	7,23E-05	2,961204711	397	1	1	1	0,009768922
	CVD3_128_TLT-2	3,653390292	148	3,574071759	105	1	3,621325727	54	1	3,524038146	51	1	3,942782184	74	0,009084741	0,000119613	3,710755157	397	1	1	1	0,00895248
E	CVD3_129_FABP4	4,506293621	148	4,749083196	105	1	4,609559387	54	1	4,896814289	51	1	5,565280108	74	1,10E-06	0,01262521	4,813874702	397	1	1	1	0,000231174
	CVD3_130_TFPI	7,813938117	148	7,815378271	105	1	7,815309488	54	1	7,815451101	51	1	8,130559739	74	0,000110996	0,000136585	7,882127306	397	1	1	1	0,000269706
	CVD3_131_PAI	5,147222513	148	5,096254688	105	1	4,942492784	54	1	5,259061411	51	1	5,768033324	74	0,003781847	0,00097622	5,267519811	397	1	1	1	0,000116873
	CVD3_132_CCL24	4,551781197	148	4,543238726	105	1	4,641783699	54	1	4,438896989	51	1	5,100092008	74	0,084619188	0,058820711	4,691064981	397	1	1	1	0,101081799
	CVD3_133_TR	3,999966982	148	4,166011384	105	1	4,073032548	54	1	4,264459563	51	1	4,335980496	74	0,195476474	1	4,122872388	397	1	1	1	1
	CVD3_134_TNFRSF10C	5,268035503	148	5,124592774	105	1	5,214672139	54	1	5,029214623	51	1	5,589927395	74	0,094438378	7,41E-05	5,287873341	397	1	1	0,662082537	0,014325967
	CVD3_135_GDF-15	3,578658997	148	4,0465052	105	0,170444667	3,917044882	54	1	4,18358083	51	0,035226356	4,187240078	74	2,23E-06	1	3,846375213	397	1	1	1	0,002695452
	CVD3_136_SELE	1,697763158	148	1,451778231	104	0,831430385	1,545848744	54	1	1,350182077	50	0,068831308	1,985668563	74	0,178981342	2,35E-05	1,696661752	397	0,036750469	1	0,005835282	0,019560569
	CVD3_137_AZU1	2,690683291	146	2,750877242	101	1	2,899988021	53	1	2,58623409	48	1	2,873863594	74	1	1	2,7549818	397	1	1	1	1
	CVD3_138_DLK-1	4,355028865	148	4,17930364	105	1	4,279340677	54	1	4,073382071	51	1	4,611582919	74	1	0,002612771	4,369312649	397	1	1	1	1
	CVD3_139_SPON1	1,143748639	147	1,652456122	105	8,26E-05	1,348673053	54	1	1,974108783	51	4,64E-08	1,407166546	74	0,000394736	1	1,367370718	396	1	1	0,000414848	1
	CVD3_140_MPO	3,737971033	148	3,751726503	105	1	3,873731794	54	1	3,62254443	51	1	3,960808871	74	1	1	3,815025087	397	1	1	1	1
	CVD3_141_CXCL16	5,543239125	148	5,566795753	105	1	5,514562948	54	1	5,622101075	51	1	5,708036422	74	0,729575287	1	5,595549935	397	1	1	1	1
	CVD3_142_IL-6RA	10,08520046	148	9,883450778	105	0,193771994	9,967995751	54	1	9,793932571	51	0,010955027	10,27486262	74	1	0,000713548	10,07521397	397	0,146715622	1	0,013453379	0,246002027
	CVD3_143_RETN	5,557511165	148	5,635072297	105	1	5,745654308	54	1	5,517985463	51	1	5,865754408	74	0,190639205	1	5,668484138	397	1	1	1	1
	CVD3_144_IGFBP-1	3,947299067	148	4,466849904	105	0,320228214	4,216363131	54	1	4,732071192	51	0,014066943	4,473380148	74	1	1	4,213217986	397	1	1	1	1
	CVD3_145_CHIT1	1,894610794	145	1,828359949	102	1	1,842967792	51	1	1,813752105	51	1	2,17068838	72	1	1	1,951876053	388	1	1	1	1
	CVD3_148_TR-AP	4,572033426	148	4,532584906	105	1	4,51307697	54	1	4,553240369	51	1	4,831019913	74	0,057352646	0,052466383	4,639155048	397	1	1	1	0,246841722
	CVD3_149_CCL22	1,456290636	147	1,484501921	102	1	1,709588648	52	1	1,250411724	50	1	1,616849562	74	1	1	1,573519921	395	1	1	0,506090623	1
	CVD3_150_PSP-D	2,124911826	123	2,137534613	73	1	2,148832558	40	1	2,123840134	33	1	2,294289454	58	1	1	2,154668765	378	1	1	1	1
	CVD3_151_PI3	2,766383105	142	2,766158163	103	1	2,745502824	52	1	2,78721851	51	1	2,955674057	73	1	1	2,823958502	362	1	1	1	1
	CVD3_152_Ep-CAM	3,661913677	148	3,701987679	105	1	3,578998261	54	1	3,832211769	51	1	3,95711638	74	1	1	3,718738001	397	1	1	1	1
	CVD3_153_AP-N	4,281121417	148	4,162454751	105	1	4,260751047	54	1	4,058376319	51	0,694650818	4,471391688	74	0,061093778	0,000202486	4,296661958	397	1	1	0,080350217	0,013804155
	CVD3_154_AXL	7,004520662	148	6,949841769	105	1	6,930243885	54	1	6,970592469	51	1	7,258507148	74	0,002884347	0,00015951	7,053263905	397	1	1	1	0,004824806
	CVD3_155_IL-1RT1	5,807138539	148	5,88023056	105	1	5,816315216	54	1	5,94790563	51	1	6,05517908	74	0,003757643	1	5,897665566	397	1	1	1	0,260798036
	CVD3_156_MMP-2	2,392057288	148	2,321862951	105	1	2,262587818	54	1	2,384624855	51	1	2,641381612	74	0,135839417	0,033293859	2,437138369	397	1	1	1	0,122980029
	CVD3_157_FAS	4,046768092	148	4,030951489	105	1	3,966660484	54	1	4,099024319	51	1	4,3623762	74	0,000480974	0,000662742	4,123214038	397	1	1	1	1,56E-05
	CVD3_158_MB	5,876464089	148	5,712041309	105	1	5,696316441	54	1	5,728691169	51	1	6,306937062	74	0,03807487	0,000243565	5,910964089	397	1	1	1	0,051409189
	CVD3_159_TNFSF13B	5,386181939	148	5,490598927	105	1	5,360366123	54	1	5,628492485	51	0,140285553	5,624154912	74	0,030307205	1	5,492425182	397	1	1	1	1
	CVD3_160_PRTN3	4,114372456	148	4,183297166	105	1	4,358884468	54	1	3,9973812	51	1	4,492007591	74	0,067153261	1	4,231277861	397	1	1	1	1
E	CVD3_161_PCSK9	1,738155831	148	1,690442832	105	1	1,674707382	54	1	1,707103897	51	1	2,029372047	74	1,43E-05	2,42E-05	1,797317993	397	1	1	1	0,002207816
	CVD3_162_U-PAR	3,554459051	148	3,734753131	105	1	3,587784877	54	1	3,890366576	51	0,028097305	3,851688781	74	0,009641371	1	3,688337429	397	1	1	1	1
	CVD3_163_OPN	4,283348288	148	4,721950604	105	0,00409135	4,444474414	54	1	5,015748924	51	1,04E-06	4,760645585	74	1,17E-05	1	4,513942882	397	1	1	0,001525509	0,580438336
	CVD3_164_CTSD	3,288937358	76	3,271412991	54	1	3,271042621	24	1	3,271709288	30	1	3,41993151	63	1	1	3,321721354	322	1	1	1	1
	CVD3_165_PGLYRP1	6,388241677	148	6,360510661	105	1	6,445293729	54	1	6,270740353	51	1	6,774863299	74	0,001027923	0,026164153	6,476971407	397	1	1	1	0,071654524
	CVD3_166_CPA1	3,589630131	148	3,490432577	105	1	3,443596598	54	1	3,540023613	51	1	3,985238239	74	1	0,016006954	3,668022023	397	1	1	1	0,654232973
	CVD3_167_JAM-A	4,1239389	148	4,282167343	105	1	4,255913374	54	1	4,309965662	51	1	4,224547008	74	1	1	4,19316863	397	1	1	1	1
	CVD3_168_Gal-4	2,556953537	148	2,591210293	105	1	2,57324733	54	1	2,610229901	51	1	3,077021104	74	3,87E-05	0,000576606	2,694507591	397	1	1	1	0,001469169
	CVD3_169_IL-1RT2	4,177114303	148	3,994806066	105	1	4,057832521	54	1	3,928072173	51	0,711711182	4,378398087	74	0,049847495	2,94E-06	4,183479168	397	0,184805957	1	0,152128769	0,001931194
	CVD3_170_SHPS-1	2,760256348	148	2,832177841	105	1	2,804685778	54	1	2,861287085	51	1	3,000256348	74	0,125719908	1	2,839769862	397	1	1	1	0,787312479
	CVD3_171_CCL15	5,973325652	148	6,123964648	105	1	6,06876359	54	1	6,182412827	51	1	6,350825652	74	0,002194063	1	6,111014841	397	1	1	1	0,148297403
	CVD3_172_CASP-3	6,925121451	148	6,926948993	105	1	6,941102432	54	1	6,911962999	51	1	6,792283614	74	1	1	6,895013343	397	1	1	1	1
	CVD3_173_uPA	4,001184934	148	4,023211961	105	1	4,034693443	54	1	4,011055099	51	1	4,208211961	74	0,163079181	1	4,063941691	397	1	1	1	1
	CVD3_174_CPB1	3,240448805	148	3,075261547	105	1	3,075716573	54	1	3,074779754	51	1	3,553624481	74	1	0,002781355	3,276056913	397	1	1	1	0,876660757
	CVD3_175_CHI3L1	4,362873817	148	4,976064937	105	0,000939057	4,585964408	54	1	5,389112556	51	7,92E-06	4,95334679	74	0,008821911	1	4,678049493	397	1	1	0,005092707	1
E	CVD3_176_ST2	3,117928314	148	3,272860103	105	1	3,242918304	54	1	3,304563184	51	1	3,67468507	74	1,35E-06	0,023539896	3,277009395	397	1	1	1	0,000249695
	CVD3_177_t-PA	4,516474981	148	4,567502652	105	1	4,533259266	54	1	4,603760355	51	1	4,96316417	74	0,011352392	0,38951449	4,637056062	397	1	1	1	0,046372512
	CVD3_178_SCGB3A2	2,120517156	148	1,799686396	105	0,972102485	1,808527666	54	1	1,790325052	51	1	2,061395534	74	1	1	2,011557696	397	1	1	1	1
	CVD3_179_EGFR	0,978593607	148	0,78857945	105	0,006961563	0,858748763	54	1	0,714282532	51	0,00255184	1,074944959	74	1	6,52E-05	0,957944959	397	0,002359542	1	0,001532445	1
	CVD3_180_IGFBP-7	3,282452788	148	3,258118167	105	1	3,252938273	54	1	3,263602761	51	1	3,638804139	74	0,000201832	0,000216866	3,36261495	397	1	1	1	0,001429674
	CVD3_181_CD93	8,719861631	148	8,698631901	105	1	8,634854124	54	1	8,766161313	51	1	8,945469739	74	0,077756286	0,025513084	8,765604874	397	1	1	1	0,038521899
	CVD3_182_IL-18BP	5,361065763	148	5,411801928	105	1	5,341886584	54	1	5,485829939	51	1	5,724984682	74	6,25E-05	0,002818491	5,458903601	397	1	1	1	0,000447506
	CVD3_183_COL1A1	1,951568589	148	1,975017109	105	1	1,813673193	54	1	2,145851843	51	1	2,072582102	74	1	1	1,994446967	397	1	1	1	1
	CVD3_184_PON3	5,468671965	148	5,116940305	105	0,093962947	5,322263057	54	1	4,899539745	51	0,002900244	5,523874668	74	1	1	5,391171965	397	1	1	0,019684683	1
E	CVD3_185_CTSZ	4,092640173	148	4,112095128	105	1	4,085835869	54	1	4,139899049	51	1	4,536491524	74	3,20E-07	3,59E-06	4,201248281	397	1	1	1	2,28E-06
	CVD3_186_MMP-3	6,282384097	148	6,212879592	105	1	6,169546259	54	1	6,258761945	51	1	6,482519232	74	1	1	6,302519232	397	1	1	1	1
	CVD3_187_RARRES2	10,88256236	148	10,95111191	105	1	10,91192672	54	1	10,99260211	51	1	11,16715695	74	0,000132478	0,114985787	10,96969749	397	1	1	1	1,64E-05
	CVD3_188_ICAM-2	4,256769419	148	4,231833769	105	1	4,177865515	54	1	4,288976627	51	1	4,5128505	74	0,034056225	0,028701292	4,315120771	397	1	1	1	0,105979421
	CVD3_189_KLK6	2,948038053	130	3,299337815	97	0,144357205	3,067905484	47	1	3,516884207	50	0,012596234	2,856035722	66	1	0,007814996	3,043123013	277	1	1	0,210708965	0,025118534
	CVD3_190_PDGF-subunit-A	2,421198958	148	2,33930771	105	1	2,231471731	54	1	2,453486982	51	1	2,747077337	74	1	1	2,465482742	397	1	1	1	1
	CVD3_191_TNF-R1	4,283302131	148	4,469273817	105	1	4,333427256	54	1	4,613111352	51	0,229512766	4,663977807	74	0,000204345	1	4,430329158	397	1	1	1	0,008912282
	CVD3_192_IGFBP-2	6,938819321	148	7,562271058	105	7,72E-08	7,301376878	54	0,504681836	7,838511954	51	1,74E-09	7,148954456	74	1	0,161514133	7,17227878	397	0,000253212	1	3,48E-07	1
	CVD3_193_vWF	4,609387637	148	4,866654046	105	1	4,748521771	54	1	4,991735279	51	1	4,996549799	74	0,076424806	1	4,779928178	397	1	1	1	1
	CVD3_194_PECAM-1	4,149363027	148	4,134030337	105	1	4,155972136	54	1	4,110797844	51	1	4,306863027	74	1	1	4,182781946	397	1	1	1	1
	CVD3_195_NT-pro-BNP	1,168315406	76	1,060069792	54	1	0,989336458	25	1	1,121046803	29	1	1,197481139	47	1	1	1,146163542	100	1	1	1	1
E	CVD3_196_CCL16	4,637836749	148	4,862952579	105	1	4,843587499	54	1	4,88345678	51	1	5,191079992	74	2,70E-06	0,48003849	4,825701613	397	1	1	1	0,005950521
	ONC2_101_TXLNA	3,387715942	144	3,44978325	104	1	3,362421131	53	1	3,540571334	51	1	3,337646498	72	1	1	3,380751163	380	1	1	1	1
	ONC2_102_VEGF-A	9,000932837	144	9,197866598	104	1	9,007801286	53	1	9,395385452	51	0,014555143	9,40440506	72	5,14E-05	1	9,127858098	380	1	1	1	0,011717996
	ONC2_103_CPE	3,545860982	144	3,352848161	104	1	3,356906579	53	1	3,34863059	51	1	3,523360982	72	1	1	3,474739895	380	1	1	1	1
	ONC2_105_KLK13	4,469188459	144	4,478654271	104	1	4,341426405	53	1	4,621263623	51	1	4,61279957	72	1	1	4,513422652	380	1	1	1	1
	ONC2_106_CEACAM1	7,179696145	144	7,130866017	104	1	7,124126176	53	1	7,137870164	51	1	7,279487811	72	1	0,416334855	7,182687627	380	1	1	1	1
	ONC2_107_MSLN	2,184451233	144	2,881678798	104	0,001893413	2,492018057	53	1	3,286620351	51	2,03E-05	2,121048456	72	1	0,007176016	2,377366749	380	0,154378495	1	0,000233496	1
	ONC2_108_TNFSF13	8,06047265	144	8,074008975	104	1	7,95124047	53	1	8,201591931	51	1	8,324500428	72	0,107897632	0,100818312	8,096642847	380	1	1	1	0,07911558
	ONC2_109_EGF	6,817341027	144	6,810722437	104	1	6,770426719	53	1	6,85259838	51	1	6,96754936	72	1	1	6,825187482	380	1	1	1	1
	ONC2_110_TNFRSF6B	3,70052298	144	4,04168751	104	0,070234776	3,762768787	53	1	4,331544222	51	0,000168918	4,168995202	72	0,001091033	1	3,897531114	380	1	1	0,011350274	0,200128279
	ONC2_111_SYND1	6,49400002	144	6,734603653	104	1	6,479331519	53	1	6,999886458	51	0,001428819	6,846430576	72	0,006020705	1	6,617556266	380	1	1	0,020385887	0,155178541
	ONC2_112_TGFR-2	6,8429005	144	6,743600286	104	1	6,67804332	53	1	6,811728114	51	1	7,198386611	72	0,004741802	6,76E-06	6,870103155	380	1	1	1	0,000137167
	ONC2_113_IL-6	2,207391793	144	2,9982091	104	0,061897816	2,70742717	53	1	3,300394244	51	0,003719651	3,231766793	72	0,000108211	1	2,654391102	380	1	1	1	1
	ONC2_114_CD48	6,369494205	144	6,309232452	104	1	6,287443628	53	1	6,33187574	51	1	6,671646982	72	0,01807469	0,000156568	6,407601556	380	1	1	1	0,003920827
	ONC2_115_SCAMP3	3,557284563	144	3,680404221	104	1	3,604310244	53	1	3,759482275	51	1	3,638534563	72	1	1	3,595655496	380	1	1	1	1

	ONC2_116_LY9	5,098852456	144	5,028986003	104	1	4,995061838	53	1	5,064240528	51	1	5,285727456	72	1	0,036619314	5,11078424	380	1	1	1	1
	ONC2_117_IFN-gamma-R1	3,712864903	144	3,659365972	104	1	3,590098918	53	1	3,731349381	51	1	3,946962126	72	0,063267767	0,00056804	3,730366822	380	1	1	1	0,003446082
	ONC2_118_ITGAV	3,113038682	144	2,950453212	104	1	2,991558074	53	1	2,907736395	51	1	3,160122015	72	1	0,917303069	3,056883833	380	1	1	1	1
	ONC2_120_TNFSF10	7,390499039	144	7,263746902	104	1	7,26204516	53	1	7,265515379	51	1	7,588276817	72	0,672354933	1,00E-04	7,393471721	380	0,603004232	1	1	0,054444046
O	ONC2_121_hK11	5,306863159	144	5,858364227	104	3,83E-05	5,566785853	53	1	6,161377048	51	1,20E-07	5,361654825	72	1	0,013489601	5,498605409	380	0,011603143	1	3,43E-06	1
	ONC2_122_GPC1	4,238733692	144	4,234855487	104	1	4,11843495	53	1	4,355841535	51	1	4,392067025	72	1	1	4,25895929	380	1	1	1	1
	ONC2_123_TFPI-2	7,128642479	144	7,551810214	104	0,236963941	7,308250708	53	1	7,804921074	51	0,000339711	7,410656368	72	0,312274176	1	7,306554158	380	1	1	0,009775906	1
	ONC2_124_hK8	6,2083035	144	6,413682773	104	1	6,218100408	53	1	6,616935036	51	0,025652821	6,143650722	72	1	1	6,259972085	380	1	1	0,011466929	1
	ONC2_125_VEGFR-2	6,892835393	144	6,742995649	104	1	6,751451745	53	1	6,734207942	51	1	6,998113171	72	1	0,000636367	6,873489168	380	0,17309163	1	1	0,277237142
	ONC2_126_LYPD3	3,955314081	144	3,884208312	104	1	3,827189081	53	1	3,943463591	51	1	4,073300192	72	1	1	3,959205656	380	1	1	1	1
	ONC2_127_PODXL	3,680091798	144	3,992858892	104	0,005270095	3,84163792	53	1	4,150010099	51	9,06E-05	3,945786243	72	0,002461377	1	3,823036857	380	1	1	0,015720092	1
	ONC2_128_S100A4	2,905447429	143	2,967449177	104	1	2,877356652	53	1	3,061074743	51	1	2,698378665	72	1	1	2,85968422	379	1	1	1	1
	ONC2_129_IGF1R	3,543038383	144	3,61748817	104	1	3,429926486	53	1	3,812405214	51	1	3,758107828	72	0,437591741	0,836995123	3,597133304	379	1	0,501512166	1	0,537691129
	ONC2_130_ERBB2	6,578419086	144	6,554759898	104	1	6,494348069	53	1	6,617540818	51	1	6,845155197	72	0,007232089	0,001292156	6,61854071	380	1	1	1	0,001222535
	ONC2_131_ERBB3	7,418911925	144	7,397512353	104	1	7,346149871	53	1	7,450889049	51	1	7,618078592	72	0,361556104	0,035692566	7,451488659	380	1	1	1	0,00551026
	ONC2_132_SCF	8,554079711	144	8,242397018	104	0,473068527	8,444115088	53	1	8,032768436	51	0,01012005	8,701510266	72	1	0,003298128	8,469255816	380	1	1	0,022592246	0,007314204
	ONC2_133_SPARC	6,017387411	144	5,967985701	104	1	5,925062987	53	1	6,012591659	51	1	6,085304077	72	1	1	6,010181303	380	1	1	1	1
	ONC2_134_GZMH	3,859637177	144	3,970887177	104	1	3,879448497	53	1	4,065911686	51	1	4,06797051	72	1	1	3,912178613	380	1	1	1	1
	ONC2_135_TGF-alpha	1,762816379	144	1,923596294	104	1	1,827845205	53	1	2,023102327	51	1	2,009899712	72	1	1	1,862158767	380	1	1	1	1
	ONC2_136_FURIN	3,532772564	144	3,656848419	104	1	3,500467794	53	1	3,819361616	51	0,327718848	3,794230897	72	0,060385666	1	3,621272027	380	1	1	0,447249417	0,039377171
	ONC2_137_CYR61	4,379355457	144	4,340381098	104	1	4,211155771	53	1	4,474674084	51	1	4,499355457	72	1	1	4,34624542	380	1	1	1	1
	ONC2_138_hK14	5,60851527	144	5,515539843	104	1	5,565770249	53	1	5,463339617	51	1	5,88164027	72	1	0,10635874	5,648248619	380	1	1	1	0,784846375
	ONC2_139_FADD	1,845019466	134	1,863761052	102	1	1,737976739	51	1	1,989545366	51	1	1,460679211	69	1	0,419993298	1,742331385	357	1	1	1	0,001847186
	ONC2_140_MetAP-2	3,250268844	144	3,19923252	104	1	3,0706331	53	1	3,332875053	51	1	3,071518844	72	1	1	3,184457764	380	1	1	1	1
[illegible]	[illegible]	[illegible]	[illegible]	[illegible]	[illegible]	[illegible]	[illegible]	[illegible]	[illegible]	[illegible]	[illegible]	[illegible]	[illegible]	[illegible]	[illegible]	[illegible]	[illegible]	[illegible]	[illegible]	[illegible]	[illegible]	[illegible]

	INF_136_MCP-4	4,293407201	147	4,171346377	102	1	4,141738534	51	1	4,20095422	51	1	4,651689514	72	0,905390347	0,011677843	4,325517353	398	1	1	1	1
	INF_137_CCL11	8,590750838	147	8,472133622	104	1	8,306364391	52	1	8,637902853	52	1	8,79337967	73	1	0,055488709	8,572254666	398	1	0,518569882	1	0,044576102
	INF_138_TNFSF14	2,693886467	145	2,830136467	104	1	2,680617237	52	1	2,979655698	52	1	2,886489207	73	1	1	2,767174139	396	1	1	1	1
	INF_139_FGF-23	3,080958105	147	3,243777964	104	1	3,11368181	52	1	3,373874118	52	1	3,425794561	73	1	1	3,206797509	398	1	1	1	1
	INF_140_IL-10RA	2,457658851	31	2,289400787	25	1	2,312543644	14	1	2,259946241	11	1	2,813178564	9	1	1	2,40786654	101	1	1	1	1
	INF_141_FGF-5	2,47330376	112	2,436885109	81	1	2,378594511	39	1	2,491012094	42	1	2,483245263	58	1	1	2,443949437	229	1	1	1	1
	INF_142_MMP-1	1,535281716	144	2,189170605	103	0,004239438	1,947994134	51	1	2,425709067	52	0,000439389	1,779033619	73	1	1	1,813479997	249	1	1	0,027768897	1
	INF_143_LIF-R	3,395979232	146	3,369028494	104	1	3,309509263	52	1	3,428547725	52	1	3,535842246	73	1	0,398144127	3,405610566	398	1	1	1	0,455955103
	INF_144_FGF-21	5,509421389	147	5,943209981	104	1	5,723209981	52	1	6,163209981	52	1	6,028088801	73	1	1	5,750573218	391	1	1	1	1
	INF_145_CCL19	9,838794815	147	9,899052533	104	1	9,826552533	52	1	9,971552533	52	1	10,05126802	73	1	1	9,871439349	398	1	1	1	1
	INF_148_IL-15RA	2,063404768	147	2,11820696	103	1	2,044957389	51	1	2,190047886	52	1	2,187579938	72	1	1	2,092950942	394	1	1	1	1
	INF_149_IL-10RB	6,932353454	147	6,968500105	104	1	6,812250105	52	1	7,124750105	52	0,640671547	7,13359099	73	0,044628904	1	6,983226316	398	1	1	1	1
	INF_150_IL-22-RA1	5,041818182	2	2,801818182	2	1	2,871818182	1	1	2,731818182	1	1	NaN	0	NA	NA	3,921818182	11	1	1	1	NA
	INF_151_IL-18R1	7,157543621	147	7,19728394	104	1	7,096418555	52	1	7,298149324	52	1	7,491223613	73	0,004940882	0,038141001	7,230564437	398	1	1	1	0,01410597
	INF_152_PD-L1	3,027995754	31	3,06606027	26	1	3,040605725	11	1	3,084726937	15	1	3,10756027	20	1	1	3,075824976	122	1	1	1	1
	INF_153_Beta-NGF	2,090850072	100	2,022664357	70	1	2,05814362	31	1	1,994462892	39	1	2,024122486	58	1	1	2,043486751	277	1	1	1	1
	INF_154_CXCL5	11,28023476	147	11,08986323	104	1	11,13197861	52	1	11,04774784	52	1	11,25670201	73	1	1	11,176998	398	1	1	1	1
	INF_155_TRANCE	4,857118945	146	4,612892127	104	1	4,704911358	52	1	4,520872896	52	1	4,703762781	73	1	1	4,775625944	398	1	1	1	1
E	INF_156_HGF	6,863292672	147	6,912954498	104	1	6,82151219	52	1	7,004396806	52	1	7,376114403	73	1,48E-06	0,000725063	6,975815796	398	1	1	1	6,46E-05
	INF_157_IL-12B	4,520505127	147	4,630830219	104	1	4,466695603	52	1	4,794964834	52	1	4,721353138	73	1	1	4,595414949	398	1	1	1	1
	INF_158_IL-24	2,928083333	6	3,026416667	6	1	3,71975	1	1	2,88775	5	1	3,23975	4	1	1	2,99025	22	1	1	1	1
	INF_159_IL-13	2,517003873	11	2,378822055	5	1	2,458822055	1	1	2,358822055	4	1	1,902155388	3	1	1	2,383558897	21	1	1	1	1
	INF_160_ARTN	1,181144338	12	0,992811005	2	1	0,967811005	1	1	1,017811005	1	1	2,767811005	1	1	1	1,389916268	22	1	1	1	1
	INF_161_MMP-10	6,887744843	147	7,028769176	104	1	6,885692253	52	1	7,171846099	52	1	7,174267069	73	1	1	7,010404577	398	1	1	1	1
OE	INF_162_IL-10	3,419344934	147	4,209011993	104	1,64E-06	3,707761993	52	1	4,710261993	52	5,23E-10	4,84968507	73	4,23E-12	1	3,97102022	397	1	0,00410586	0,529576635	0,065101325
	INF_163_TNF	2,271555556	15	1,864888889	5	1	1,772222222	3	1	2,003888889	2	1	2,542222222	6	1	1	2,24	18	1	1	1	1
	INF_164_CCL23	10,24572917	147	10,37508094	104	1	10,25921556	52	1	10,49094633	52	1	10,31418131	73	1	1	10,29830957	398	1	1	1	1
	INF_165_CD5	4,720278196	147	4,589508311	104	1	4,532681388	52	1	4,646335234	52	1	4,828447984	73	1	0,138693779	4,700350116	398	1	1	1	1
	INF_167_Flt3L	9,716142154	147	9,726620961	104	1	9,654313269	52	1	9,798928653	52	1	9,815720013	73	1	1	9,728782982	398	1	1	1	1
	INF_168_CXCL6	8,031044004	147	8,041881262	104	1	7,809958185	52	1	8,273804339	52	1	7,971386004	73	1	1	7,988686963	398	1	1	1	1
	INF_169_CXCL10	8,754774047	147	9,398252592	104	0,00021799	9,1434449	52	1	9,653060284	52	5,93E-05	9,420167766	73	0,000656802	1	9,068462715	398	0,281301544	1	0,035053641	0,578277697
	INF_170_4E-BP1	6,630052717	147	6,311210488	104	1	6,261787411	52	1	6,360633565	52	1	6,292266863	73	1	1	6,434117111	398	1	1	1	1
	INF_171_IL-20	2,114426788	22	2,922790425	10	1	4,183290425	4	1	2,082457091	6	1	2,595790425	6	1	1	2,398839205	27	1	1	1	1
	INF_172_SIRT2	5,307710339	146	5,088424249	104	1	4,919578095	52	1	5,257270403	52	1	4,87620349	73	1	1	5,126344593	396	1	1	1	1
	INF_173_CCL28	2,298657641	85	2,192882965	59	1	2,181569406	24	1	2,200640834	35	1	2,244454021	52	1	1	2,251408855	115	1	1	1	1
	INF_174_DNER	7,916663461	147	7,678396193	104	0,01528401	7,818492346	52	1	7,538300039	52	4,03E-05	7,926400671	73	1	0,079080105	7,846175222	398	1	1	0,001046932	1
O	INF_175_EN-RAGE	3,577056095	127	4,513901031	101	4,87E-08	4,533758457	50	0,000153701	4,494432967	51	7,40E-05	3,661946336	66	1	0,000572107	3,976062649	398	0,342001882	1	1	0,064914312
	INF_176_CD40	9,592672753	147	9,682256741	104	1	9,558795202	52	1	9,805718279	52	1	9,813589723	73	1	1	9,648440979	398	1	1	1	1
	INF_177_IL-33	2,292843137	3	2,518176471	5	1	2,771176471	2	1	2,349509804	3	1	2,339509804	9	1	1	2,383823529	4	1	1	1	1
	INF_178_IFN-gamma	2,187493216	80	2,154925234	57	1	2,086900825	23	1	2,200941745	34	1	2,249415513	37	1	1	2,187825287	177	1	1	1	1
	INF_179_FGF-19	8,587225654	147	8,797657366	104	1	8,897657366	52	1	8,697657366	52	1	8,79215157	73	1	1	8,678641436	398	1	1	1	1
	INF_180_IL-4	2,931619981	9	2,71050887	3	1	2,66550887	2	1	2,80050887	1	1	2,94800887	4	1	1	2,8593324	63	1	1	1	1
	INF_181_LIF	2,248354978	14	1,891212121	4	1	1,666212121	2	1	2,116212121	2	1	2,421212121	2	1	1	2,157121212	48	1	1	1	1
	INF_182_NRTN	2,28812782	15	2,155339941	11	1	2,333294486	4	1	2,053651629	7	1	2,173651629	7	1	1	2,206223058	57	1	1	1	1
	INF_183_MCP-2	9,043271002	147	9,074850018	104	1	8,962926941	52	1	9,186773095	52	1	9,419894802	73	0,100099453	0,619848086	9,119029959	398	1	1	1	0,209062735
	INF_184_CASP-8	3,1010356	145	3,401357662	103	1	3,276954464	51	1	3,523368491	52	1	2,581168807	73	0,316628273	1,58E-05	3,036802557	398	1	1	1	2,32E-06
	INF_185_CCL25	6,940468742	147	6,730800374	104	1	6,710704221	52	1	6,750896528	52	1	7,066468183	73	1	0,130229765	6,873514952	398	1	1	1	1
	INF_186_CX3CL1	6,693210151	147	6,675687911	104	1	6,611457142	52	1	6,73991868	52	1	6,962078849	73	0,062450704	0,039589652	6,724810736	398	1	1	1	0,061721124
	INF_187_TNFRSF9	7,018678451	147	7,185633579	104	1	6,933229733	52	1	7,438037426	52	0,000225979	7,278769775	73	0,0215249	1	7,110031766	398	1	1	0,00486456	0,723008016
	INF_188_NT-3	2,531967401	144	2,501294324	104	1	2,539563555	52	1	2,463025093	52	1	2,540237949	73	1	1	2,510854764	388	1	1	1	1
	INF_189_TWEAK	9,690810807	147	9,47295301	104	0,074922561	9,51670301	52	1	9,42920301	52	0,037932616	9,861191946	73	1	0,000268452	9,654810325	398	0,22767891	1	0,130550645	0,120035028
	INF_190_CCL20	6,561795268	147	7,12973025	104	0,614083264	6,881172557	52	1	7,378287942	52	0,240199525	7,226009228	73	0,003808618	1	6,861734285	398	1	1	1	1
	INF_191_ST1A1	2,744644903	101	2,642110721	84	1	2,565826114	43	1	2,722116528	41	1	2,327705959	48	0,215336657	1	2,586174512	297	1	1	1	1
	INF_192_STAMPB	5,459575821	147	5,236727836	104	1	5,13057399	52	1	5,342881682	52	1	5,230012873	73	1	1	5,323831693	398	1	1	1	1
	INF_193_IL-5	3,24078877	15	3,034122103	11	1	3,084122103	7	1	2,946622103	4	1	3,943010992	9	1	1	3,19548574	51	1	1	1	1
	INF_194_ADA	5,999021369	147	5,917104834	104	1	5,957104834	52	1	5,877104834	52	1	6,02787143	73	1	1	5,965223153	398	1	1	1	1
	INF_195_TNFB	4,291592793	147	4,188476624	104	1	4,200207393	52	1	4,176745855	52	1	4,374245855	73	1	1	4,292965201	398	1	1	1	1
	INF_196_CSF-1	8,266263375	147	8,403303537	104	0,966975415	8,283399691	52	1	8,523207383	52	0,008155878	8,470309596	73	0,018419041	1	8,345515075	398	1	1	0,48242998	1
