# Supplementary material for: A two-step strategy for identification of plasma protein biomarkers for endometrial and ovarian cancer
Source: Clin Proteomics. 2018 Dec 1;15:38. doi: 10.1186/s12014-018-9216-y (PMC6271635; doi:10.1186/s12014-018-9216-y)
Supplement: Supplementary file 2 — Additional file 2: Table 2. CA125 values in the clinical analyses and the PEA NPX values for some of the benign and ovarian cancer cohorts used in the discovery analysis. [file 12014_2018_9216_MOESM2_ESM.pdf]

Supplementary Table 2

	CA 125 Arbitrary unit		CA 125 NPX	
	**Benign tumors cohort I**	**Ovarian cancer cohort I**	**Benign tumors cohort I**	**Ovarian cancer cohort I**
Mean	73,74	1059,96	3,51	5,98
Median	16,3	204,15	3,38	6,25
Range	2.9-4631.6	6.2-14880.2	0.74-7.38	1.90-9.03
SD	402,38	2333,14	1,30	1,81
P-value vs benign	NA	< 2,2e-16	NA	< 2,2e-16
